# Design and Synthesis of Transferrable Macro‐Sized Continuous Free‐Standing Metal‐Organic Framework Films for Biosensor Device

**DOI:** 10.1002/advs.202310189

**Published:** 2024-03-11

**Authors:** Zhe Zhao, Xinyi Ke, Jiayuan Huang, Ziyu Zhang, Yue Wu, Gaoshan Huang, Ji Tan, Xuanyong Liu, Yongfeng Mei, Junhao Chu

**Affiliations:** ^1^ Department of Materials Science & State Key Laboratory of Molecular Engineering of Polymers Fudan University Shanghai 200438 P. R. China; ^2^ College of Biological Science and Medical Engineering Donghua University Shanghai 201620 P. R. China; ^3^ Shanghai Frontiers Science Research Base of Intelligent Optoelectronics and Perception, Institute of Optoelectronics Fudan University Shanghai 200438 P. R. China; ^4^ Yiwu Research Institute of Fudan University Yiwu Zhejiang 322000 P. R. China; ^5^ International Institute of Intelligent Nanorobots and Nanosystems Fudan University Shanghai 200438 P. R. China; ^6^ State Key Laboratory of High Performance Ceramics and Superfine Microstructure, Shanghai Institute of Ceramics Chinese Academy of Sciences Shanghai 200050 P. R. China

**Keywords:** formation mechanism, free‐standing metal organic framework film, glucose sensor, molecular adsorption, transferred device

## Abstract

Metal organic framework (MOF) films have attracted abundant attention due to their unique characters compared with MOF particles. But the high‐temperature reaction and solvent corrosion limit the preparation of MOF films on fragile substrates, hindering further applications. Fabricating macro‐sized continuous free‐standing MOF films and transferring them onto fragile substrates are a promising alternative but still challenging. Here, a universal strategy to prepare transferrable macro‐sized continuous free‐standing MOF films with the assistance of oxide nanomembranes prepared by atomic layer deposition and studied the growth mechanism is developed. The oxide nanomembranes serve not only as reactant, but also as interfacial layer to maintain the integrality of the free‐standing structure as the stacked MOF particles are supported by the oxide nanomembrane. The centimeter‐scale free‐standing MOF films can be transferred onto fragile substrates, and all in one device for glucose sensing is assembled. Due to the strong adsorption toward glucose molecules, the obtained devices exhibit outstanding performance in terms of high sensitivity, low limit of detection, and long durability. This work opens a new window toward the preparation of MOF films and MOF film‐based biosensor chip for advantageous applications in post‐Moore law period.

## Graphical abstract

A universal process for preparing transferable free‐standing metal organic framework (MOF) films by using the induction and the bonding effects of oxide nanomembranes is designed. Mass production of centimeter‐scale free‐standing MOF films is realized, and the growth mechanism of free‐standing film is studied in detail. It is demonstrated that the device obtained by transferring MOF film on a flexible substrate possesses excellent real‐time sensing ability.

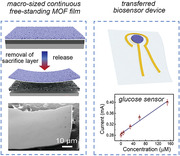

## 1 Introduction

Metal organic framework (MOF) films have attracted vast interests due to their abundant characters compared with MOF particles, and they have various applications including sensors, energy storage devices, and catalysts.^[^ ^1^, ^2^, ^3^, ^4^, ^5^ ^]^ Several approaches have been developed to fabricate MOF layer by integrating MOF crystals with certain substrates.^[^ ^6^, ^7^, ^8^ ^]^ In our group, we also adopted an effective technique to prepare MOF films on substrates by engaging an induction effect of oxide nanomembranes deposited by atomic layer deposition (ALD), which leads to growth of MOF films with good adhesion, uniformity, and conformality.^[^ ^9^, ^10^, ^11^, ^12^, ^13^, ^14^, ^15^ ^]^ However, the high temperature of ALD and severe corrosion of solvents in the wet chemistry process may harm the fragile substrate, and the conformal growth of MOF films of this ALD‐based induction approach hinders the single‐surface growth on substrate with complex geometry. Especially in the post‐Moore law period, MOF can hardly act as active materials in on‐chip device, because the strict preparation conditions could damage the integrated circuit and even the whole device. Hence, the approach which includes preparing free‐standing MOF films and then transferring them onto target substrates has become an ideal strategy to handle above‐mentioned challenges. Some pioneered researches have reported the efforts in preparing free‐standing MOF films. For instance, Mao et al.^[^ ^16^ ^]^ fabricated large scale, robust, and well intergrown free‐standing HKUST‐1 membranes from copper hydroxide nanostrand films and applied it on gas separation. Zhang et al.^[^ ^17^ ^]^ demonstrated a facile technique for preparing free‐standing MOF film with size as large as centimeter scale by using anodized aluminum oxide as aluminum precursor. Kim et al.^[^ ^18^ ^]^ fabricated free‐standing MOF patterns by a micro‐confined interfacial synthesis approach. Kubo et al.^[^ ^19^ ^]^ used a spray‐dry technique to prepare HKUST‐1/carbon nanotube composite, which can be peeled off to form a free‐standing film. Researchers also tried to prepare more stable free‐standing MOF film via connecting MOF particles by using polymers (e.g., poly(methyl methacrylate)).^[^ ^20^, ^21^, ^22^ ^]^ Generally, a dense and firm MOF film with macro‐scale dimension is essential for the assembly of the MOF film‐based device. However, this kind of MOF films can hardly be produced by previous strategies.^[^ ^23^, ^24^ ^]^ Those free‐standing MOF films are relatively loose and mechanically fragile, and they may not survive in the post‐treatments. The robust polymer‐strengthened film, on the other hand, may suffer from the insulated polymer which prevents the effective charge transfer in the film.^[^ ^23^ ^]^ Thus, universal strategy to fabricate macro‐sized continuous free‐standing MOF films with less unnecessary material is desired. More fantastic applications of such free‐standing MOF films in advanced devices and chips could be expected on the basis of deep understanding of the formation mechanism.

Herein, to handle these challenges, we developed a universal strategy to prepare free‐standing MOF films, and they can be transferred onto versatile substrates for device applications. In this strategy, sacrificial layer, interfacial layer, and induction layer were deposited on the surface of substrate prior to the assembly of MOF film, and production of free‐standing MOF film with tunable dimensions was realized by releasing the film upon the selective removal of the sacrificial layer. The formation mechanism of the free‐standing MOF films is studied with the help of specific microstructural characterizations. We found that the induction layer helps to produce MOF structure, while the interfacial layer is served as connection link to maintain the structural integrity, leading to formation of transferrable film with area larger than 2 cm^2^. Delicate flexible devices which are practically impossible to be prepared by using previous approaches are fabricated on fragile substrates. The fabricated glucose sensor by this “transfer” approach demonstrates an ultra‐high sensitivity of 4840 µA mM^−1^ cm^−2^ and a low limit of detection (LOD) of 0.14 µM. The corresponding device fabricated with transferrable MOF film by means of photolithography can be pasted on human skin to achieve an impressive glucose sensing performance. On the basis of the structural property, theoretical simulation proves that the free‐standing MOF film with low adsorption energy possesses the advantage of capturing target molecules. We believe that the current strategy of preparing macro‐sized continuous free‐standing MOF films open a new window for MOF‐related structures and can be applied in integratable devices for various interesting on‐chip applications. Although the cost and procedure could be weak points, the gradual maturation of the approach and parallel fabrication of devices will effectively reduce the cost of individual device, making the current approach promising in the future practical applications.

## 2 Results and Discussion

### 2.1 Release and Transfer of Robust Free‐Standing MOF Film

**Figure 1:**
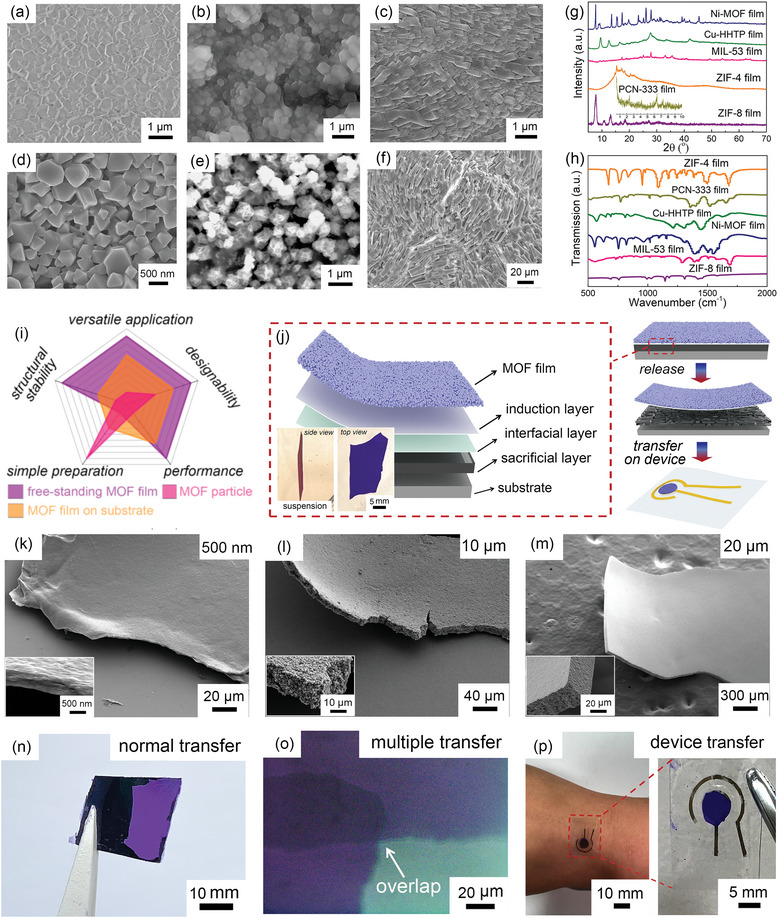
Fabrication and transfer of the free‐standing MOF film. a–f) SEM images of versatile MOF films on Si wafer prepared by an ALD‐oxide nanomembrane induction strategy: a) ZIF‐67 film, b) ZIF‐8 film, c) ZIF‐4 film, d) MIL‐53 film, e) PCN‐333 film, and f) Ni‐MOF film. g) XRD patterns and h) FTIR spectra of the resultant MOF films. i) Comparison of free‐standing MOF film, MOF film on substrate, and MOF particle. j) The fabrication and transfer process of the free‐standing MOF film. The insets are top and side views of a centimeter‐scale free‐standing MOF film suspended in the water. k) SEM image of the thin free‐standing MOF film (≈500 nm). The inset is the image with high magnification. l) SEM image of the mid free‐standing MOF film (≈10 µm). The inset is the image with high magnification. m) SEM image of the thick free‐standing MOF film (20 µm). The inset is the image with high magnification. n) Photograph of the free‐standing MOF film transferred onto the thin graphite paper. o) Optical microscopy image of the stacked MOF films with multiple transfer process. p) Photograph of the free‐standing MOF film transferred for sensor device which can adhere onto human skin.

In our previous researches, several MOF films have been fabricated with the induction effect of the ALD‐oxide nanomembrane,^[^ ^9^, ^10^, ^14^, ^15^ ^]^ and the approach is developed to be universal for more MOF films. SEM images of typical MOF films like ZIF‐67, ZIF‐8, ZIF‐4, MIL‐53, PCN‐333, and Ni‐MOF films are shown in **Figure** **1**a–f. X‐ray diffraction (XRD) and Fourier transform infrared (FTIR) spectroscopy characterizations were carried out to confirm the formation of the MOF films (Figure 1g,h). The universality of this strategy thus provides an excellent foundation for fabrication of various free‐standing MOF films. Compared with MOF particle and traditional MOF film on substrate, free‐standing MOF film shows extraordinary advantages toward broad applications, designability, structure stability, and good performance (Figure 1i). However, effective preparation of transferrable macro‐sized free‐standing MOF film is still challenging. Here, on the basis of aforementioned fabrication of uniform and robust MOF films, we mainly choose ZIF‐67 as the typical example due to the stable zeolite structure to investigate fabrication process and growth mechanism of the transferrable free‐standing MOF film.

In order to peel off the MOF film from the substrate to generate firm and stable free‐standing film, we designed a composite sample structure, and three additional layers were deposited on substrate surface prior to the assembly of MOF film, as shown in Figure 1j. The first layer from bottom to top is sacrificial layer, which survives high temperature and harsh chemical environment, and can be removed easily to release above film. Here, poly(3,4 ethylenedioxythiophene)/poly(styrenesulfonate) (PEDOT/PSS) film is served as the sacrificial layer due to its stable structure and high solubility in water. The second layer is the interfacial layer which is chemically inert during the fabrication process and can help to maintain the intact of the transferrable MOF film, while the third layer is the induction layer which reacts to form MOF film. The whole macro‐sized continuous free‐standing MOF film can be peeled off and transferred onto versatile target substrates. To choose the suitable materials for the interfacial layer, we have tested several oxide nanomembranes. As shown in Figure S1 (Supporting Information), it can be clearly seen that both ALD‐TiO_2_ and ALD‐Al_2_O_3_ nanomembranes can somehow induce the assembly of MOF films, but ALD‐Al_2_O_3_ nanomembrane provides weaker effect because scattered aggregation of MOF particles can be observed. Hence, an Al_2_O_3_ nanomembrane is chosen as interfacial layer, and the thickness of ALD‐Al_2_O_3_ nanomembrane is set to be 200 cycles due to the large electric resistivity of Al_2_O_3_. In addition, since the MOF film induced by ALD‐TiO_2_ nanomembrane exhibits poor attachment with the substrate — MOF film is removed after ultrasonication process (Figure S2, Supporting Information), it may not be suitable as the induction layer. According to our previous reports,^[^ ^9^, ^11^, ^14^ ^]^ ALD‐ZnO nanomembrane is an ideal induction layer to assemble MOF film. As shown in Figure S3 (Supporting Information), we prepared MOF patterns from patterned ZnO nanomembrane, and this further demonstrates the excellent induction effect of ZnO nanomembrane. Therefore, we choose ALD‐ZnO nanomembrane as the induction layer. To optimize the thickness of induction layer, we studied the influence of ZnO nanomembrane thickness. As shown in Figure S4 (Supporting Information), with thin induction layer, MOF film exhibits poor adhesion with substrate, and cracks can be observed. With the thickness increases to 300 cycles, dense MOF film is obtained. Hence, the optimized thickness of ALD‐ZnO nanomembrane is determined to be 300 cycles. After the formation of MOF film with the induction effect of ZnO nanomembrane, the sacrificial PEDOT/PSS film was removed by water, and ALD‐oxide nanomembranes confined the MOF particles to generate an intact transferrable free‐standing MOF film (Figure 1j). Here, optical image of a piece of smooth centimeter‐scale continuous free‐standing ZIF‐67 film fabricated by this strategy is shown in the insets of Figure 1j and Figure S5a (Supporting Information). It is worth noting that although the support of Al_2_O_3_ nanomembrane remarkably promotes the mechanical stability of the ZIF‐67 film, the fraction of Al_2_O_3_ in the film can be as low as ≈0.53% (Supporting Note 1), which is neglectable. In order to investigate the chemical stability of the resultant free‐standing MOF film, we immersed the MOF film into alkaline solution (0.1 M NaOH) for 24 h. As shown in Figure S5b (Supporting Information), no obvious morphological change can be noticed, indicating a great chemical stability of the free‐standing MOF film.

The formation process of the uniform MOF film is carefully investigated. Generally, the process can be described into two steps:^[^ ^25^ ^]^ nucleation on substrate and further growth of the crystals. The thickness of MOF film is decided by the growth step, and solution with very high concentration will hinder growth of MOF, producing a thinner layer.^[^ ^26^, ^27^, ^28^ ^]^ Thus, prolonging the aging time and diluting the solution to decrease the effect of self‐limitation should be beneficial to the growth process, leading to the formation of a thicker film. As shown in Figure 1k–m and Figure S6 (Supporting Information), the thickness of free‐standing MOF films is precisely controlled in the range of ≈500 nm – ≈50 µm. Typical examples of a thin film of ≈500 nm (Figure 1k), a mid‐film of ≈10 µm (Figure 1l), as well as a thick film of ≈20 µm (Figure 1m) are demonstrated with high resolution. In the corresponding enlarged SEM images (insets in Figure 1k–m), it can be seen that close stack of the MOF particles form the resultant MOF films, which are completely different from normal individual MOF particles prepared in solution (Figure S7, Supporting Information). Moreover, the size of MOF particles in the film was also tunable. Enhance the concentration of organic linker is beneficial to the fast crystal nucleation but negative to the crystal growth,^[^ ^29^ ^]^ which leads to small particle size. On the other hands, reducing the concentration of organic linker is good for crystal growth which is preferable for large size. As shown in Figure S8 (Supporting Information), the average particle size in the free‐standing MOF film can be controlled between ≈50 nm to ≈2 µm. In addition to prepare MOF films with controllable thickness and particle size, the current strategy is also capable of mass production (Figure S9a,b, Supporting Information), and the robustness of MOF film can even achieve self‐standing behavior (Figure S9c, Supporting Information). In our experiment, the size of the free‐standing MOF film can reach a few square centimeters (Figure S10, Supporting Information), and such large MOF film is convenient in the following handling and transferring processes for advanced device application on target substrate.

To further illustrate the universality of this strategy toward more MOF structures, we additionally prepared free‐standing ZIF‐8 film, ZIF‐4 film, MIL‐53 film, and conductive Cu‐HHTP film (Figure S11, Supporting Information). The optical images and SEM images show dense and smooth MOF films, proving the generality of this strategy. Here, an interesting phenomenon was observed. The free‐standing MOF films with centimeter scale suspended in the solution is normally flat (inset of Figure 1j) while they exhibit a slightly rolled geometry in most cases after being dried, suggesting the existence of internal strain gradient. With the help of dynamic simulation (Supporting Note 2), the predicted geometry is shown in the inset of Figure S12a (Supporting Information), which fits well with the experimental result. On the basis of theoretical simulation, the strain and stress distributions along the thickness of the film are also specifically illustrated in Figure S12b (Supporting Information). Moreover, the location of an artificially bent free‐standing MOF film with a small curvature radius of ≈4 mm was further characterized and uniform and dense structure without crack can be observed (Figure S13, Supporting Information), indicating a good mechanical stability of the nanomembrane‐supported MOF film. Due to the unique structure of macro‐sized continuous free‐standing MOF films, they can be transferred onto many substrates for various purposes. As shown in the Figure S14 (Supporting Information), the macro‐sized free‐standing MOF films are pasted onto the surfaces of some substrates commonly used in the fields of electrochemistry, flexible electronics, biomedicine, optoelectronics such as glassy carbon, polydimethylsiloxane (PDMS) film, Eco‐flex film, rubber, and indium tin oxide conductive glass with the help of Nafion. This demonstrates the universal possibility of transferring free‐standing MOF films with good integrity onto substrates where direct growth of MOF films is challenging. Furthermore, Figure S15 (Supporting Information) shows a transferred MOF film onto thin graphite paper (20 µm), and good contact between MOF film and graphite paper can be observed. Even when the MOF film is bent with the thin graphite paper, uniform structure without any crack is noticed (Figure S16, Supporting Information), which is beneficial for the application. More, I‐V curve of transferred MOF film on thin graphite paper was measured (Figure S17, Supporting Information) and the calculated high conductivity is noticed, proving the advantages of current transfer approach and good contact between the film and the substrate.

In addition, the free‐standing MOF films can be transferred with different manners, such as normal transfer, curved transfer, and multiple transfer. As shown in the Figure 1n, the free‐standing MOF film is directly transferred onto the surface of thin graphite paper and is fixed by Nafion, and this manner is suitable for electrochemical devices. For electronic device, a hetero/homo transfer is also important. As shown in the Figure 1o, two pieces of free‐standing MOF films are overlapped in a multiple transfer process, which may be beneficial to the formation of on‐chip junction device. Figure 1p shows that the free‐standing MOF film is transferred onto a sensor device, completely covers the circular gold electrode, and acts as a working electrode. The detailed schematic of the sensor device is demonstrated in Figure S18 (Supporting Information). The whole device is fabricated on the surface of PDMS film due to its flexibility and biological affinity, and device can adhere to the surface of human skin (Figure 1p).

**Figure 2:**
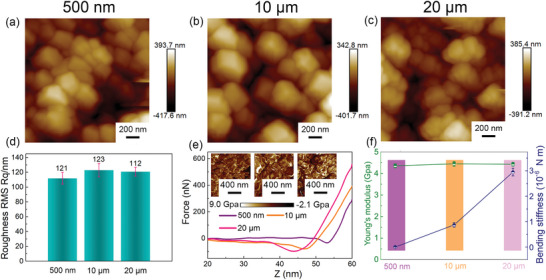
Morphology and tomography of the free‐standing MOF films. AFM images of the free‐standing MOF films with a) small thickness, b) mid thickness, and c) large thickness. d) The roughness of the free‐standing MOF films with various thicknesses. e) The force‐displacement curves of the free‐standing MOF films with various thicknesses. Insets are mappings of DMT moduli of the free‐standing MOF films with various thicknesses. f) The calculated Young's moduli and bending stiffnesses of the free‐standing MOF films with various thicknesses.

The morphology and mechanical properties of free‐standing MOF films transferred onto Si wafer are specifically investigated by atomic force microscope (AFM). Here, surface topographies were recorded over a scanned areas of 4 µm^2^ (**Figure** **2**a–c). No obvious morphology change can be observed with the increasing film thickness. Also, the height‐position curves of various MOF films are shown in Figure S19 (Supporting Information), indicating relative flat surfaces. The 10 µm film exhibits a largest roughness of 123 nm, and the 20 µm film shows a smallest roughness of 112 nm (Figure 2d). Since the size of MOF particles in this case is ≈200 nm, all the MOF films with various thicknesses exhibit a roughness smaller than the particle size, implying a smooth and dense structure. In addition, mechanical properties of MOF films were also measured at nanoscale by using AFM, and the corresponding force‐distance curves are shown in Figure 2e. With the increasing thickness, the displacement under the same force decreases. This is because that the thinner MOF films are more flexible, and the brittleness of MOF film increases with the thickness. Moreover, the Derjaguin, Muller, and Toporov (DMT) model has been used for evaluating the mechanical properties.^[^ ^19^ ^]^ Maps of elastic moduli of free‐standing MOF films calculated from the DMT model are shown in the insets of Figure 2e. The results illustrate that Young's modules are in the order of GPa. The average Young's moduli and bending stiffnesses of free‐standing MOF films were calculated and the results are shown in Figure 2f, where a constant Young's modulus of ≈4.4 Gpa is noticed. With the increased thickness, the bending stiffness of MOF film increases, due to the rigid character of MOF. The thick free‐standing MOF film exhibits the highest bending stiffness up to 2×10^−6^ Nm, implying a firm and stable structure.

### 2.2 Formation Mechanism of Free‐Standing MOF Film

**Figure 3:**
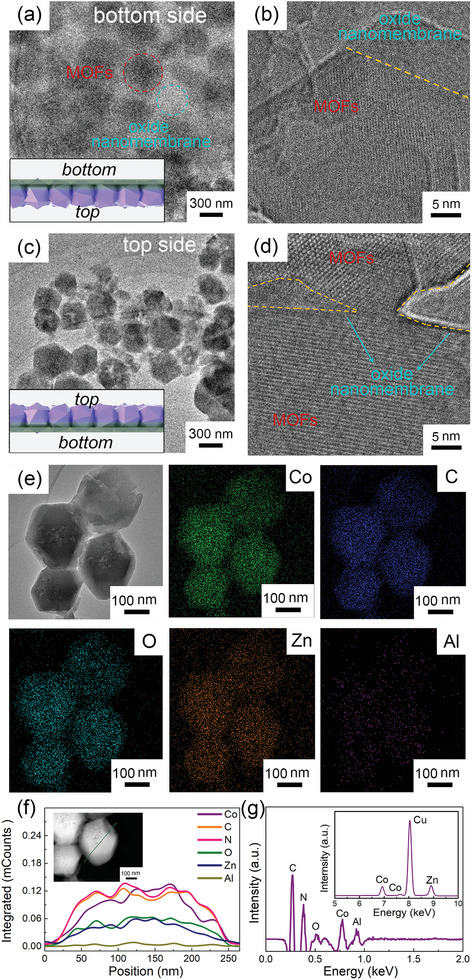
Formation mechanism of the free‐standing MOF film. a) and b) Cryo‐TEM images of free‐standing MOF film (bottom side view) with low and high magnifications. The inset is corresponding schematics. c) and d) Cryo‐TEM images of free‐standing MOF film (top side view) with low and high magnifications. The inset is corresponding schematics. e) Cryo‐TEM image of the free‐standing MOF film with elemental mapping results. f) Linear EDS scan along the dashed line in the inset, demonstrating the distribution of the elements in the free‐standing MOF film. Inset is the TEM image with high magnification. g) EDS spectrum of the free‐standing MOF film in (e). The inset is the high energy region of the spectrum.

In order to go deeper into the structure of free‐standing MOF films, the sample was characterized by Cryo‐Transmission Electron Microscopy (Cryo‐TEM) to reduce the damage and the results are shown in **Figure** **3**. In the experiment, the free‐standing MOF film was fixed on a 360° sample holder, and both sides of film can be observed. As shown in Figure 3a, the morphology of the bottom side of MOF film shows that a vaguer membrane‐like structure covers on the surface of particles. This confirms that continuous MOF film consists of MOF particles and oxide nanomembrane which supports them. The phenomenon can be further proved in TEM image with high magnification (Figure 3b), where amorphous ALD‐oxide nanomembrane supporting crystal MOF particles can be observed. We consider that partial ZnO nanomembrane (i.e., induction layer) transforms into target MOF structure, while almost all Al_2_O_3_ nanomembrane (i.e., interfacial layer), which is amorphous due to the ALD growth process, has been preserved to maintain the structural integrity (inset of Figure 3a). We also investigate the morphology of top side of the free‐standing MOF film (Figure 3c,d). MOF particles with polyhedral shape can be clearly observed in Figure 3c with low magnification, and the close stack of them builds the major structure of continuous MOF film (inset of Figure 3c). High magnification image in Figure 3d again proves that the crystal polyhedrons are supported by the amorphous oxide nanomembrane. The fast Fourier transform of TEM image of the free‐standing MOF film is shown in Figure S20 (Supporting Information), and the thon rings and diffraction spots therein imply that both crystal and amorphous structures co‐exist in the free‐standing MOF film. To further evaluate the composition of MOF film, TEM image with energy dispersive spectroscopy (EDS) mapping are shown in Figure 3e. The obtained EDS results clearly demonstrate that Co, C, O, Zn, and Al are evenly distributed over the surface of the film, implying the uniformity of the film. In addition, radial linear EDS scan along a single MOF particle (inset of Figure 3f) is shown in Figure 3f, and a typical EDS spectrum is demonstrated in the Figure 3g. The high Co, N, Zn, Al, O, and C concentrations confirm the co‐existence of MOF particles and oxide nanomembranes. On the basis of above morphological and compositional characterizations, we thus conclude that in the free‐standing MOF film, ALD‐ZnO nanomembrane as induction layer leads to formation of target MOF particles, and ALD‐Al_2_O_3_ nanomembrane as interfacial layer helps to reinforce the free‐standing structure. MOF particles stack with each other and supported by the oxide nanomembrane to form the intact MOF film.

### 2.3 Structure Characteristics of the Free‐Standing MOF Film

**Figure 4:**
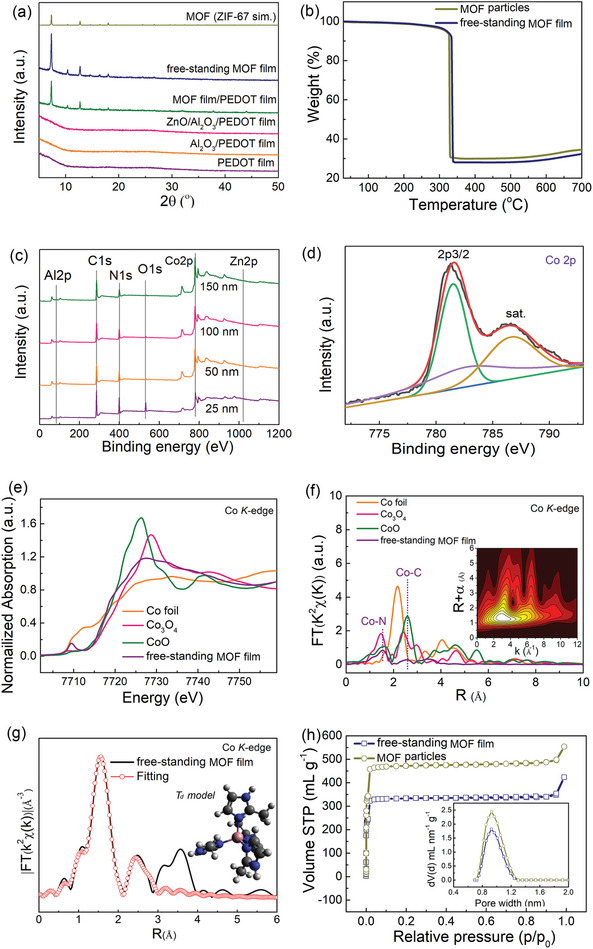
Structural characterizations of the free‐standing MOF film. a) XRD patterns of PEDOT film, Al_2_O_3_/PEDOT film, ZnO/Al_2_O_3_/PEDOT film, MOF film/PEDOT film, and free‐standing MOF film. Simulated patterns of MOF (ZIF‐67 (sim.)) is also shown for comparison. b) TGA curve of free‐standing MOF film compared with that of MOF particles. c) XPS survey scans at various depths of the free‐standing MOF film. d) High resolution Co 2p_3/2_ spectrum of the free‐standing MOF film. e) XANES spectra and f) FT k_3_‐weighted EXAFS spectra of the free‐standing MOF film, CoO, Co_3_O_4_, and Co foil. Inset demonstrates a WT analysis of the free‐standing MOF film. g) Fitting result of the FT‐EXAFS for the free‐standing MOF film at Co K‐edge. Inset of (g) shows a T_d_ model of ZIF‐67 structure. h) Nitrogen adsorption‐desorption isotherms of the free‐standing MOF film and the MOF particles. Inset demonstrates the corresponding pore size distributions.

In order to further study the structure of macro‐sized continuous free‐standing MOF films, XRD patterns of the film and related samples are shown in **Figure** **4a**. It can be observed that the PEDOT/PSS film shows an amorphous nature (plot: PEDOT film), and after deposition of oxide nanomembranes, no obvious peak is demonstrated, again demonstrating the amorphous structure of ALD‐oxide nanomembranes (plots: Al_2_O_3_/PEDOT film and ZnO/Al_2_O_3_/PEDOT film). For MOF film prepared by the induction process, sharp peaks emerge and all the peaks well conformed to the simulated pattern, proving the formation of MOF layer (plot: MOF film/PEDOT film).^[^ ^30^, ^31^ ^]^ After removing the sacrificial PEDOT film to release the MOF film, the free‐standing MOF film exhibits same crystal structure. In addition, since temperature is an important factor which highly effects the structure stability, thermogravimetric analysis (TGA) of free‐standing MOF film and MOF particles were carried out to study the thermostability of the samples. As shown in Figure 4b, when the temperature is increased to 300 °C, a gradual drop of mass caused by oxidation degradation of organic linkers can be seen. It worth noting that the crashing temperature of free‐standing MOF film is relatively higher (≈6 °C) than that of MOF particles, and this phenomenon is ascribed to the protection from oxide nanomembranes that enhances the thermal stability. The elemental composition and chemical state of free‐standing MOF film were then investigated by using X‐ray photoelectron spectroscopy (XPS). The results confirm the presence of Co, N, C, O, Al, and Zn (Figure 4c). With the increase of depth, the intensity of every elements signal remains unchanged, as the sample is stable and homogeneous (Figure 4c). The high‐resolution Co 2p_3/2_ spectrum can be deconvoluted into 3 peaks at 781.6, 782.8, and 787.0 eV, corresponding to Co^3+^, Co^2+^, and satellite species, respectively (Figure 4d),^[^ ^24^ ^]^ indicating the existence of active Co ion components. The high‐resolution C 1s, O 1s, and N 1s spectra are shown in Figure S21 (Supporting Information), and it can be clearly observed that C─C and C─N bonds from imidazole (MI) ligand exist in the free‐standing MOF film. For the XPS spectrum of N 1s, the co‐existence of C = N and oxidized‐N peaks demonstrates that partial H atoms adjacent to the N atoms are replaced by the O atoms.^[^ ^32^, ^33^, ^34^ ^]^ In order to probe the composition of the sample for more details, we also carried out inductive coupled plasma (ICP) mass spectrometry analysis, and the results show high Co concentration and relatively low Al and Zn concentrations in the composite (Figure S22, Supporting Information).

Moreover, X‐ray absorption near‐edge structure (XANES) spectra were obtained to provide insights into the electronic structure and coordination environment of the Co atoms in the film (Figure 4e). It can be seen that the Co k‐edge XANES of free‐standing MOF film is very different compared with those of Co_3_O_4_. In addition, the corresponding absorption edge of the free‐standing MOF film is located between those of CoO and Co foil, indicating that Co in the free‐standing MOF film carries a positive charge and the valence of the Co atom is situated both Co^0^ and Co^2+^. Extended X‐ray absorption fine structure (EXAFS) spectra of free‐standing MOF film, CoO, Co_3_O_4_, and Co foil in k space are shown in Figure S23 (Supporting Information), and all the samples exhibit high intensity.^[^ ^30^ ^]^ The Fourier‐transformed (FT) k_3_‐weighted EXAFS spectra (Figure 4f) of the free‐standing MOF film show two main peaks at 1.55 and 2.57 Å, corresponding to Co‐N and Co‐C coordination shells, respectively. In order to clearly distinguish the backscattering atoms, we performed wavelet transform (WT) analysis for the k_3_‐weighted EXAFS signal (Figure S24, Supporting Information and inset of Figure 4f) in view of the powerful resolution afforded by this method both in radial distance and k‐space. Free‐standing MOF film shows only one WT maximum at 3.2 Å^−1^ (inset of Figure 4f), which is identical to the CoPc (Figure S24, Supporting Information), implying the existence of Co‐N/C. On the other hand, no Co‐Co coordination can be observed.^[^ ^35^, ^36^ ^]^ According to previous reports, target MOF (ZIF‐67) have two structure: T_d_ geometry (inset of Figure 4g) and O_h_ geometry (Figure S25, Supporting Information).^[^ ^37^ ^]^ To further confirm its concrete structure, the quantitative coordination configuration of the Co atom in the free‐standing MOF film are obtained by EXAFS fitting, as shown in Figure 4g and Table S1 (Supporting Information). The experimental total Co‐N/C coordination number and the Co‐N/C average bond length of the free‐standing MOF film are very close to those of T_d_ geometry and the values reported in the literature.^[^ ^37^, ^38^ ^]^

The property of MOF is closely connected with their pore structures. Here, nitrogen adsorption‐desorption isotherms and corresponding pore size distributions of free‐standing MOF film and pristine MOF particles prepared in solution were investigated, and the results are shown in Figure 4h. The MOF particles exhibits a very high specific surface area of 1982 m^2^ g^−1^, while the free‐standing MOF film shows a similar isotherm with a large surface area of 1393 m^2^ g^−1^. The specific surface area of free‐standing MOF film is relatively smaller than that of MOF particles, but the value remains very large. We believe that the oxide nanomembrane support and the stack of MOF particles in the film may lead to the slight decrease. In addition, type I isotherms exist in both free‐standing MOF film and MOF particles (Figure 4h), implying the existence of microporous structure in the samples.^[^ ^28^, ^39^ ^]^ The pore size distributions in the samples are also demonstrated in the inset of Figure 4h, and open micropore peak located at ≈0.9 nm can be clearly observed.

### 2.4 Sensing Performance of the Transferred MOF Film

**Figure 5:**
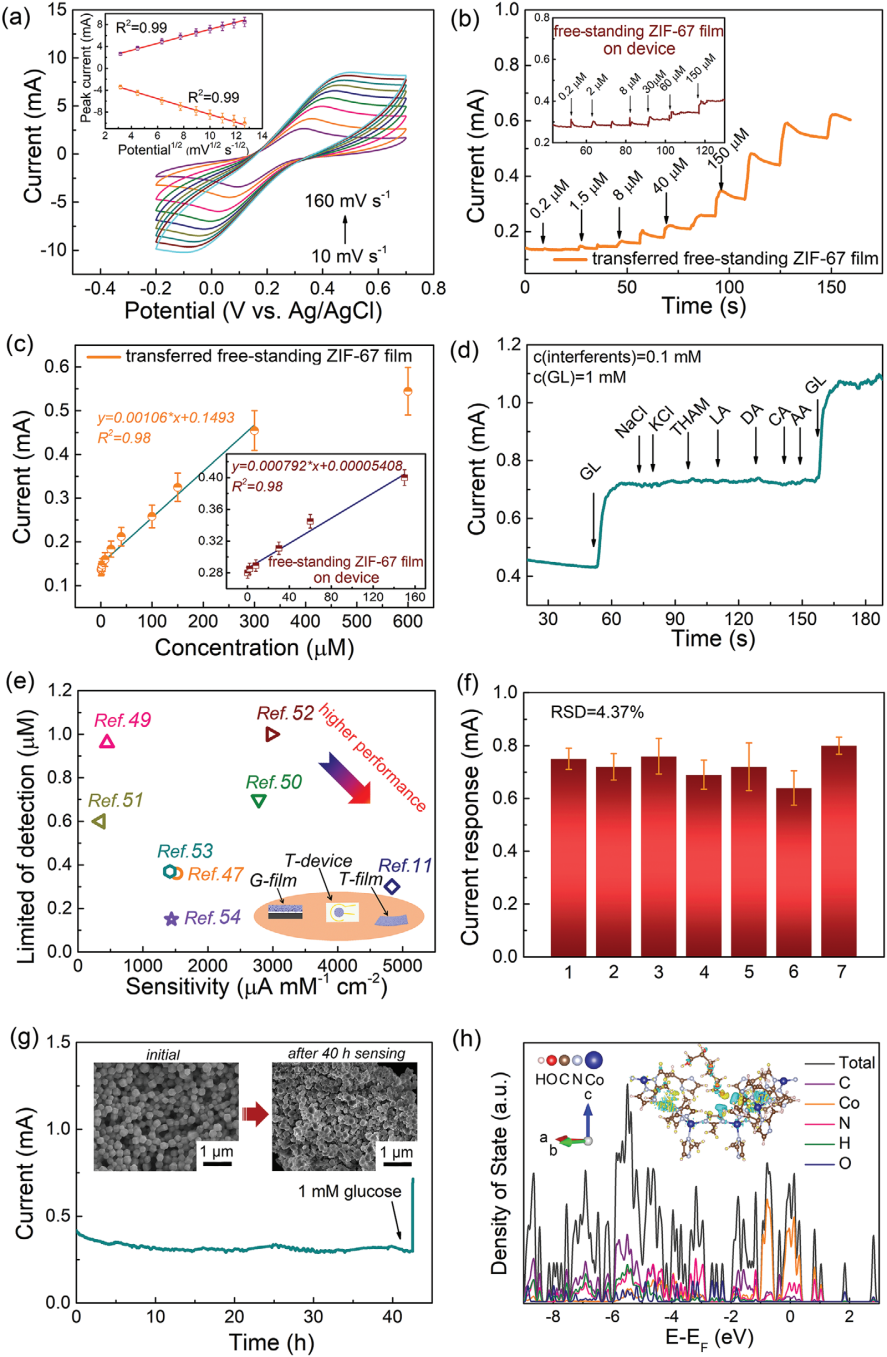
Sensing performance and mechanism of the transferred MOF film‐based device. a) CV scans at various scan rates of 10–160 mV s^−1^ in 0.1 M PBS (pH = 7.2) containing 5 mM K_3_[Fe(CN)_6_] of the transferred free‐standing ZIF‐67 film on thin graphite paper. Inset is the oxidation peak current and the reduction peak current as functions of square root of the scan rate, derived from (a). b) *I–t* curve of the transferred free‐standing ZIF‐67 film with the successive addition of glucose in 0.1 M NaOH at a potential of 0.6 V. Inset shows the sensing performance of the device consisting of free‐standing ZIF‐67 film. c) Calibration plot derived from (b). Inset is the calibration plot of the device. d) *I–t* curve of the transferred free‐standing MOF film for the continuous addition of 1.0 mM glucose, 0.1 mM NaCl, 0.1 mM KCl, 0.1 mM THAM, 0.1 mM LA, 0.1 mM DA, 0.1 mM CA, 0.1 mM AA, and 1 mM glucose at 0.6 V. e) Summary of glucose sensing performance of MOF‐based structure reported in literature published in recent years. ZIF‐67 film grown on regular thick graphite paper (denoted as G‐film), transferred ZIF‐67 film (denoted as T‐film), and ZIF‐67 film‐based device (denoted as T‐device) are marked with orange pattern. f) Statistics of the current responses of the transferred free‐standing ZIF‐67 films when 1 mM of glucose is added for 7 times. g) *I–t* curve of the transferred free‐standing ZIF‐67 film in 0.1 M NaOH with 1.0 mM glucose at 0.6 V. 1 mM glucose was added to the solution after 40 h to test the performance recovery. Inset is the SEM images of free‐standing ZIF‐67 film before and after continuous sensing test of 40 h. h) DOS of the free‐standing ZIF‐67 film after adsorbing glucose. Inset is the charge density for single molecule adsorption of glucose.

Diabetes has become one of the major diseases threatening human health, and continuous glucose levels monitoring of patient plays an important role in glycemic control. Up to now, practical evaluation of human glucose content is still based on the collection of blood, which brings pain and suffering to the body.^[^ ^39^, ^40^, ^41^ ^]^ Plenty of researches have shown that the concentration of glucose in sweat is closely related to that in blood, and thus sensing of glucose in sweat is quite promising.^[^ ^42^, ^43^, ^44^ ^]^ In this circumstance, the sensor aims to perform high sensitivity and flexibility. In addition, the interference of substrate for electrochemical sensing should be suppressed to reduce the background current and to enhance the sensitivity.^[^ ^45^, ^46^ ^]^ According to our previous investigation, ZIF‐67 film has good performance toward glucose sensing.^[^ ^11^ ^]^ So, we transfer free‐standing ZIF‐67 film onto thin graphite paper to achieve glucose sensing, because MOF films can hardly grow on fragile thin graphite paper directly. To investigate the electrochemical activity of the transferred free‐standing MOF film quantitatively, the active area of the composites was evaluated by using K_3_[Fe(CN)_6_] as a probe. As shown in **Figure** **5a**, both oxidation and reduction peaks can be observed in the cyclic voltammetry (CV) scans of the transferred free‐standing ZIF‐67 film at different scan rates, and as the scan rate rises from 10 to 160 mV s^−1^, the peak currents for oxidation and reduction also increase. The Randles‐Sevcik equation can be used to calculate the active area (A) of electrode materials:^[^ ^14^ ^]^ I_peak_ = (2.69×10^5^)n^3/2^AD^1/2^Cv^1/2^, where n is the number of transferred electrons, D and C are the diffusion coefficient and the bulk concentration of K_3_[Fe(CN)_6_], separately. The calculated A is 0.173 cm^2^ for the transferred free‐standing ZIF‐67 film, according to the slope of the peak current versus the square root of the scan rate (inset of Figure 5a). It is obvious that the transferred free‐standing ZIF‐67 film has a large active area, and this can greatly enhance the contact of active species in electrolyte and corresponding interfacial electron transfer, thereby enhancing the electrochemical performance.^[^ ^14^, ^47^ ^]^

CV scan was used to investigate the sensing property of the transferred free‐standing ZIF‐67 film. As a preliminary demonstration, Figure S26 (Supporting Information) shows the CV scans of the transferred free‐standing ZIF‐67 film electrode at a scan rate of 20 mV s^−1^ in a potential range of −0.1–0.8 V with and without 1 mM glucose in the electrolytes. Two reduction peaks at ≈0.45 and ≈0.13 V and one broad oxidation peak at ≈0.3 V are noticeable. Attention should be paid that the current responses of reduction and oxidation peaks are remarkably enhanced with 1 mM glucose, suggesting that the transferred film is capable of catalyzing the oxidation of glucose to generate additional current.^[^ ^11^, ^47^ ^]^ The current‐time (*I–t*) measurement was also utilized to assess the performance of the electrochemical sensing. Figure S27 (Supporting Information) depicts the effect of applied potential on the amperometric response of 1 mM glucose to establish the optimal potential for glucose sensing. Under 0.6 V, the transferred film exhibits an outstanding and stable response. As a result, the non‐enzymatic glucose sensing performance is quantitatively evaluated in this work at 0.6 V. We compare the sensing performance of the transferred free‐standing ZIF‐67 films with various thicknesses. As shown in the Figure S28a (Supporting Information), thin film with thickness of 500 nm exhibits unsatisfied current response toward 1 mM glucose due to the existence of thick oxide nanomembrane which is not fully transformed into target MOFs. This can be further elucidated by the results presented in Figure S29 (Supporting Information), and one can clearly see that the performance increases with the film thickness for thin films. However, thick MOF film with a thickness of 20 µm also exhibits a diminished performance due to the limitation of mass transfer and electric signal transmission. Obviously, MOF film with a moderate thickness of ≈10 µm exhibits the highest current response (Figure S28b, Supporting Information). Thus, we choose the free‐standing ZIF‐67 film of ≈10 µm for optimal glucose sensor. Specifically, Figure 5b illustrates the *I–t* curve of the sensor (≈10 µm film) with the successive addition of glucose in 0.1 M NaOH at a potential of 0.6 V. With the addition of glucose, apparent and rapid current responses can be seen, indicating a good real‐time sensing ability. The electrochemical sensing performance of the practical device (Figure 1p) is shown in the inset of Figure 5b, which also demonstrates a similar fast and apparent response toward glucose. In addition, the calibration plots correspondingly derived from *I–t* curves are shown in Figure 5c and its inset. The calibration plot shows that the free‐standing film has an ultrahigh sensitivity of 4840 µA mM^−1^ cm^−2^ and a linear range of 0.5–300 µM (linear regression equation: Y = 0.00106X+0.1493, correlation coefficient (R^2^) = 0.98). Furthermore, the LOD is calculated by using the equation: LOD = 3б/S, where б is the standard deviation (here refer to the noise of the experimental data) and S is the sensitivity, and thus a LOD of 0.14 µM is obtained. In the case of device, a large sensitivity of 4035 µA mM^−1^ cm^−2^ with a low LOD of 0.18 µM was noticed, indicating an outstanding sensing performance in real circumstance.

For comparison, as shown in Figure S30 (Supporting Information), neat glassy carbon do not show any current response. Oxide nanomembrane is a dense structure, and no active site can be used for biosensing. Thus, no current response can be observed. Figure S31 (Supporting Information) shows CV curve of ZIF‐67 particles in 0.1 M NaOH electrolyte, and no obvious oxidation and reduction peaks can be observed. With the addition of 1 mM glucose, the area surrounded by CV curve is slightly increased, indicating a negligible electrochemical activity.^[^ ^10^ ^]^ In addition, we also directly grew MOF films on thick graphite paper (thickness: ≈1 mm) with the induction effect of ALD‐oxide nanomembrane. As shown in the Figure S32 (Supporting Information), target MOF particles stacked with each other to form a dense film on the substrate. The thick substrate generates a large background current, leading to a deteriorated sensing performance. As shown in Figure S33 (Supporting Information), the film direct grows on thick graphite paper demonstrates a lower sensitivity (3203.6 µA mM^−1^ cm^−2^) and a higher LOD (0.19 µM) compared with the transferred film and corresponding sensor device. The 51% sensing performance enhancement proves that the utilization ratio of the active materials has been improved. This further confirms that the current transferring technique can achieve a good contact between MOF film and substrate and take the advantage of the target substrate.

For assessing the performance of the sensor system, the anti‐interference capability is equally critical. Figure 5d shows the *I–t* curve of the transferred film with the continuous addition of 1 mM glucose, 0.1 mM NaCl, 0.1 mM KCl, 0.1 mM tromethamine (THAM), 0.1 mM lactic acid (LA), 0.1 mM dopamine (DA), 0.1 mM citric acid (CA), 0.1 mM ascorbic acid (AA), and 1.0 mM glucose, and the result demonstrates that the interferents produce little current response compared to glucose, proving the good anti‐interference ability. As shown in Figure S34 (Supporting Information), the *I–t* curve of the transferred free‐standing ZIF‐67 film with consecutive introduction of 1 mM glucose and 3 mM interferents (e.g., NaCl, KCl, THAM, LA, CA, and DA) illustrates the considerable current response with the addition of target glucose. When adding a number of interferents, one can only observe a tiny response, with the exception of a small response from DA, elucidating good selectivity of transferred free‐standing MOF film toward the target glucose molecule. It is worth mentioning that despite concentrations of interferents being three times greater than target glucose concentration, insignificant current responses from interferents are seen, suggesting great potential of the transferred film in practical applications. We should stress that the sensing performance can compare favorably with other MOF‐based electrochemical glucose sensors in previous reports,^[^ ^11^, ^47^, ^49^, ^50^, ^51^, ^52^, ^53^, ^54^ ^]^ and the detailed comparison is summarized in Figure 5e. In Table S2 (Supporting Information), comparison of the performances of the current sensors with those of devices made from other 2D materials also confirms the impressively high performance of the transferred MOF film. To evaluate the sensing performance of the transferred free‐standing ZIF‐67 film toward large concentration of target glucose, CV scan of the sample in the range of −0.1–0.8 V with various glucose concentrations are shown in Figure S35a (Supporting Information). With the increase of glucose concentration up to 12 mM, the reduction peaks at ≈0.45 and ≈0.13 V and the oxidation peak at ≈0.3 V are significantly strengthened, implying additional current provided by oxidation of glucose. As shown in Figure S35b (Supporting Information), the calibration plots exhibit large slope, indicating a high sensitivity. Even at a high glucose concentration, the sensitivity of transferred free‐standing ZIF‐67 film is as high as 102.7 µA mM^−1^ cm^−2^. In addition, the reproducibility of the sensor is evaluated by sensing 1 mM glucose with the same sensor for 7 times, and the sensor was washed thoroughly with water after each test. As shown in Figure 5f, the similar response current with a relative standard deviation (RSD) of 4.37% proves the good reproducibility of the sensor due to the easy adsorption‐desorption process toward glucose molecules. Long‐term stability is another important parameter for sensors. Here, long‐term performance of the transferred free‐standing MOF film was evaluated at 0.6 V in 0.1 M NaOH containing 1 mM glucose. As shown in the Figure 5g, it can be clearly seen that the current response is stable over 40 h without significant deterioration, demonstrating a great long‐term stability of the free‐standing MOF film electrode. In addition, because of its excellent recovery capability, the transferred film still displays an obvious current response when 1 mM glucose is added after the evaluation of 40 h. Correspondingly, the insets of Figure 5g show the morphologies of the free‐standing ZIF‐67 film before and after 40 h sensing test, and no obvious changes can be observed, illuminating an outstanding stability.

### 2.5 Mechanism on Enhanced Sensing Mechanism

We consider that the outstanding sensing property of the free‐standing MOF film can be attributed to its unique structural properties. First, the continuous MOF film provides a conductive pathway for ion transfer. Second, the homogeneous film guarantees uniform dispersion of active sites and alleviates the aggregation, leading to maximized activity. Furthermore, the large surface area of MOF film makes it easier for target molecules to access the catalytic sites. More importantly, we notice that the electrochemical sensing performance of free‐standing ZIF‐67 film may be related to the molecule activities on the surface, and the strong adsorption toward target glucose may greatly enhance the sensing performance. Generally, the sensing of glucose molecule consists of two steps: capture of the target glucose molecule and oxidation of the glucose molecule to generate current signal.^[^ ^47^ ^]^ According to our previous investigation, the selectivity of the sensor should be determined by the single‐molecule adsorption, while the sensitivity may be decided by the multi‐molecule adsorption.^[^ ^15^ ^]^ Here, calculations of the adsorption energy based on density functional theory (DFT) were performed to gain more insight of the high performance.^[^ ^55^ ^]^ The structure model of free‐standing MOF film (side and top views) is shown in Figure S36 (Supporting Information), and the adsorption of glucose is demonstrated in Figure S37 (Supporting Information). When the potential was set at 0.6 V, the adsorption energy of glucose is calculated to be −1.64 eV. Such a low adsorption energy implies a strong capture ability toward glucose, which is beneficial to electrochemical sensing. To further determine the adsorption mechanism toward glucose, density of state (DOS) of the free‐standing ZIF‐67 film after adsorbing glucose were calculated and the results are shown in Figure 5h. Compared with the calculated DOS of bare free‐standing ZIF‐67 film (Figure S38, Supporting Information), two new peaks appear at ≈1.8 and ≈2.8 eV after adsorption, which are related to the chemical bond of O and H.^[^ ^56^, ^57^, ^58^ ^]^ The peak at low energy may originate from hydrogen bond interaction, and the peak at high energy is ascribed to the O─H covalent bond in the adsorbed glucose.^[^ ^58^ ^]^ Hence, the adsorption of glucose is enhanced due to the effect of hydrogen bond. The corresponding distribution of charge densities is shown in the inset of Figure 5h and Figure S39 (Supporting Information), and the blue and yellow regions indicate electron depletion and accumulation, respectively. The larger electron cloud and the greater charge transfer indicate higher absolute value of molecular adsorption energy in the case of glucose. Therefore, more glucose molecules are absorbed and contribute to the response current.

## 3 Conclusion

In conclusion, a universal strategy of preparing centimeter‐scale continuous free‐standing MOF film has been developed. Oxide nanomembranes is first deposited on sacrificial layer via ALD, and then partial oxide nanomembrane is transformed into target MOF materials. With the removal of the sacrificial layer, free‐standing MOF film linked by residual oxide nanomembrane is generated. Due to the unique structure where oxide nanomembrane supports MOF particles, the free‐standing MOF film has outstanding structural stability, which can be transferred onto different target substrates for various applications like electrochemical sensing. The transferred ZIF‐67 film exhibits an ultra‐high sensitivity of 4840 µA mM^−1^ cm^−2^ with a low LOD of 0.14 µM toward glucose sensing. Also, an all in one flexible sensor device is assembled by transfer process, and a high sensing performance is also demonstrated. We believe that the universal strategy of preparing various free‐standing MOF films is promising for the preparation of advantageous MOF film‐based integratable devices and chips.

## 4 Experimental Section

### Materials

Cobalt nitrate hexahydrate (Co(NO_3_)_2_·6H_2_O), 2‐MI, anhydrous ferric chloride (FeCl_3_), 1,4‐Dicarboxybenzene (H_2_BDC), benzene‐1,4‐dicarboxylic acid (C_8_H_6_O_4_), Cu(CH_3_COO)_2_·H_2_O (≥97%), 2,3,6,7,10,11‐hexahydroxytriphenylene (HHTP, ≥97%), dopamine hydrochloride (≥97%), ascorbic acid (≥99.99%), and Nafion (5 wt.%) were purchased from Aladdin Ltd. (Shanghai, China). D(+)‐Glucose monohydrate (AR, ≥99.7%), NaOH (AR, ≥99.7%), NaCl (AR, ≥99.7%), KCl (AR, ≥99.7%), potassium ferricyanide (K_3_[Fe(CN)_6_], AR, ≥99.7%), zinc acetate dihydrate (Zn(CH_3_COO)_2_·2H_2_O, AR, ≥99.7%), and lactic acid (AR, ≥99.7%) were obtained from Sinopharm Chemicals. Methanol (AR, ≥99.5%), N,N‐dimethylformamide (DMF, AR, ≥99.5%), and ethanol (AR, ≥99.7%) were purchased from Titan Ltd. PEDOT/PSS (1.3 wt. % dispersion in H_2_O, conductive grade) and MI were purchased from Sigma–Aldrich. Graphite paper, PDMS film, indium tin oxide conductive glass, and slide were purchased from Sigma–Aldrich. The solutions used for the electrochemical activity measurements were prepared by using 0.1 M PBS, pH 7.2 unless otherwise noted. The DI water used throughout all experiments was purified through a Millipore system. All the reagents were used as received without further purification.

### Preparation of Sacrificial Layer

The sacrificial layer in the current work is prepared on a slide substrate. Then a uniform PEDOT/PSS layer with a thickness of ≈10 µm was spin‐coated on the slide substrate, and finally solidified at 90 °C for 6 h.

### Al_2_O_3_ Nanomembrane Deposited by ALD

Al_2_O_3_ nanomembrane was deposited on the sacrificial layer by using ALD technology. The deposition of Al_2_O_3_ nanomembrane was performed at 120 °C in a homemade reactor. Trimethylaluminium (TMA) and DI water were used as precursors. A typical ALD cycle included TMA pulse (60 ms), waiting time (2 s), N_2_ purge (30 s), DI water pulse (60 ms), waiting time (2 s), and N_2_ purge (30 s). In the current study, Al_2_O_3_ nanomembrane with 200 ALD cycles (≈18 nm) was deposited on substrate.

### ZnO Nanomembrane Deposited by ALD

ZnO nanomembrane was deposited on Al_2_O_3_ nanomembrane by using ALD technology. The deposition of ZnO nanomembrane was performed at 150 °C in a homemade reactor. Diethylzinc (DEZ) and DI water were used as precursors. A typical ALD cycle included DEZ pulse (70 ms), waiting time (2 s), N_2_ purge (30 s), DI water pulse (50 ms), waiting time (2 s), and N_2_ purge (30 s).

### Fabrication of MOF Film (ZIF‐67 as example) on Sacrificial Layer

Co(NO_3_)_2_·6H_2_O (1.45 g) was dissolved in the mixture of methanol (40 mL) and ethanol (40 mL) to form solution A. 2‐MI (1.65 g) was dissolved in the mixture of methanol (40 mL) and ethanol (40 mL) to form solution B. ALD‐Al_2_O_3_ and ALD‐ZnO nanomembranes‐coated PEDOT/PSS was then placed into a beaker containing solution A. The beaker was sealed at 70 °C for 12 h. After solution A was cooled down to room temperature, solution B was added, and the mixture was aged at room temperature for another 24 h. After that, the composite film was taken out and washed with ethanol. The sample was subsequently dried in vacuum at 60 °C for 12 h. This process led to the formation of a uniform ZIF‐67 film.

### Peeling of the Free‐Standing MOF Films

In order to prepare free‐standing MOF film, the PEDOT/PSS as a sacrificial layer was dissolved in DI water, and then the MOF film was released which can be transfer onto other substrates.

### Synthesis of Other Free‐Standing MOF Films

Free‐standing ZIF‐8, ZIF‐4, MIL‐53, and conductive Cu‐HHTP films were also synthesized for comparison. Zn(CH_3_COO)_2_·2H_2_O (2.7 g) was dissolved in methanol (40 mL) to form solution C. 2‐MI (6 g) was dissolved in methanol (112 mL) to form solution D. ALD‐coated PEDOT/PSS was then placed into solution C, and solution D was added after 15 min. The mixture was aged at room temperature for another 24 h. After that, the sample was taken out and washed with ethanol. Then the free‐standing ZIF‐8 film was peeled off by using DI water.

Zn(CH_3_COO)_2_·2H_2_O (1.65 g) was dissolved in methanol (80 mL) to form solution E. MI (1.65 g) was dissolved in methanol (80 mL) to form solution F. ALD‐coated PEDOT/PSS was then placed into a beaker containing solution E, and solution F was added after 12 h. The mixture was aged at room temperature for another 24 h. After that, the sample was taken out and washed with ethanol. Then the free‐standing ZIF‐4 film was peeled off by using DI water.

Anhydrous ferric chloride (FeCl_3_, 1.62 g) was dissolved in DMF (100 mL) to form solution G. H_2_BDC (1.66 g) was dissolved in DMF (100 mL) to form solution H. The ALD‐coated PEDOT/PSS was then placed into a beaker containing solution G. The beaker was sealed at 150 °C for 24 h. After cooling to room temperature, solution H was added, and the mixture was sealed at 150 °C for an additional 24 h. After that, the sample was taken out and washed with ethanol. Then the free‐standing MIL‐53 films were peeled off by using DI water.

A mixture of HHTP (32.4 mg), Cu(CH_3_COO)_2_·H_2_O (10.5 mg), and DMF (2.5 mL) was prepared and sealed in a 15 mL glass vial. ALD‐coated PEDOT/PSS was placed in the glass vial. The vial was then heated at 85 °C for 12 h. After the vial was cooled down to room temperature, the sample was taken out and washed with ethanol. Then the free‐standing Cu‐HHTP film was peeled off by using DI water.

### Synthesis of the MOF Powder (ZIF‐67 as example)

MOF powder was also synthesized for comparison. The solutions A and B were mixed with vigorous stir for 5 min, and then aged in a beaker at room temperature for 24 h. The product was centrifuged, washed with ethanol for 3 times, and finally dried at 60 °C for 12 h in vacuum.

### Synthesis of MOF Film Transferred Sensor Device

A 2×4 cm PDMS film was cleaned and spinned with photoresist AZ5214. After the photoresist dried at 90 °C, the electrode was prepared by ultraviolet lithography and Au deposition (50 nm) by E‐beam evaporation. The PDMS with the electrode was treated by oxygen plasma and the free‐standing ZIF‐67 film was transferred onto the device. The device is pasted on the surface of human skin and connected with electrochemical work station by wire. The flow chart of the preparation is shown in Figure S18 (Supporting Information).

### Structural Characterizations

The morphologies of the samples were characterized by field‐emission SEM (Gemini 560, Zeiss) and Cryo‐TEM (Thermo Scientific Talos F200C 200 kV at 77 K). XRD patterns were measured by an X'Pert Pro X‐ray diffractometer equipped with Cu Kα radiation (λ = 0.1542 nm) at a current of 40 mA and a voltage of 40 kV. The EDS (Oxford X‐Max 80T) was utilized to analyze the composition of the sample. XPS analyses were made with a VG ESCALAB 220I‐XL equipment. The curve fitting of all XPS spectra was accomplished by using XPS Peak 4.1 software. TGA was performed with an STA449F3 (NETZSCH, Germany) instrument in air atmosphere with a heating rate of 10 °C min^−1^. Electrical properties of the sample were measured by a semiconductor parameter analyzer (Keithley 4200). All force measurements were made by using an Asylum Research MFP‐3D‐BIO (Santa Barbara, CA) AFM and PNP‐TR‐50 silicon nitride cantilevers (NanoWorld, Switzerland) with nominal spring constants of 0.32 N m^−1^ and 351 half‐angle openings. The EXAFS and XANES at the Co K‐edge were collected at the beamline BL11B station of the Shanghai synchrotron radiation facility. The Co K‐edge XANES data were recorded in a fluorescence excitation mode by using a Lytle detector and Co foil, CoO, and Co_3_O_4_ were used as the references. The storage ring was operating at the energy of 2.5 GeV with a maximum current of 250 mA. All XAFS spectra were analyzed using the Demeter software package (University of Chicago). The XANES raw data were normalized by a procedure consisting of several steps. The photon energy was first calibrated based on the Co 4f peak of a freshly sputtered cobalt foil which was used as a base line to set the pre‐edge at zero. Then, the spectra were normalized to yield an edge‐jump to one. For each fitting, the theoretical curved‐wave backscattering amplitude (F_j_(k)), phase‐shift functions (ϕ_j_(k)), and mean free path (Å(l)) of all the paths were calculated by using FEFF8.2 code. A Quadrasorb adsorption instrument (Quantachrome Instruments) was used to perform the nitrogen sorption/desorption measurements. The specific surface area was calculated by using single‐point Brunauer‐Emmett‐Teller (BET) method. The pore size distributions were calculated from nitrogen adsorption data by using Barrett Joyner Halenda (BJH) method provided by Quantachrome data reduction software ASiQwin Version 4.01.

### Theoretical Simulation

The dynamic simulation of free‐standing MOF film was realized by Abaqus, and the strain gradient were obtained. On the other hand, glucose molecule adsorption was simulate by using the first principle calculation on the basis of periodic DFT. A generalized gradient approximation within the Perdew‐Burke Ernzerh of exchange correction function was adopted. The wave functions were constructed from the expansion of plane waves with an energy cutoff of 500 eV. Gamma‐centered k_piont_ of 2×2×1 have been used for geometry optimization. The consistence tolerance for the geometry optimization was set as 1.0×10^−5^ eV atom^−1^ for total energy and 0.05 eV Å^−1^ for force, respectively. In order to avoid the interaction between the two surfaces, a large vacuum gap of 15 Å has been selected in the periodically repeated slabs. The adsorption energy E_ads_ was calculated according to the standard formula: E_ads_ = E_Total_‐E_Cat_‐E_Molecular_, where E_Total_ is the total energy, E_Cat_ is the catalyst energy, and E_Molecular_ is the molecular energy.

### Electrochemical Characterizations

The electrochemical glucose sensing tests were evaluated on a Zennium X (Zahner Instrument, Germany) with three‐electrode configuration. In this experiment, an Ag/AgCl (in saturated KCl solution) was used as the reference electrode and a graphite rod was used as the counter electrode. Transferred free‐standing MOF films on both thin and thick graphite papers were tailored into rectangles with the area of 10×20 mm^2^ and directly used as the working electrode. MOF powder modified glassy carbon (GC, diameter: 3 mm) was used as the working electrode for comparison. Bare GC was first polished to a mirror successively by using 1.0, 0.3, and 0.05 µm alumina slurry, and then sonicated alternately in ethanol and deionized water for several times. The active materials ink was prepared by sonicating the mixture of 4.0 mg sample, 970 µL ethanol, and 30 µL Nafion (5 wt.%) for 40 min. Next, 10 µL of the dispersion ink was dropped onto the well‐polished GC electrode and then dried under ambient conditions.

## Conflict of Interest

The authors declare no conflict of interest.

## Supporting information

Supporting Information

## Data Availability

The data that support the findings of this study are available from the corresponding author upon reasonable request.
