# Supplementary material for: Design and Synthesis of Transferrable Macro‐Sized Continuous Free‐Standing Metal‐Organic Framework Films for Biosensor Device
Source: Adv Sci (Weinh). 2024 Mar 11;11(23):2310189. doi: 10.1002/advs.202310189 (PMC11187891; doi:10.1002/advs.202310189)
Supplement: Supplementary file 1 — Supporting Information [file ADVS-11-2310189-s001.pdf]

ADVANCED SCIENCE Open Access

Supporting Information

for *Adv. Sci.*, DOI 10.1002/advs.202310189

Design and Synthesis of Transferrable Macro-Sized Continuous Free-Standing Metal-Organic Framework Films for Biosensor Device

Zhe Zhao, Xinyi Ke, Jiayuan Huang, Ziyu Zhang, Yue Wu, Gaoshan Huang, Ji Tan, Xuanyong Liu, Yongfeng Mei* and Junhao Chu*

Supporting Information

Design and Synthesis of Transferrable Macro-Sized Continuous Free-Standing Metal-Organic Framework Films for Biosensor Device

Zhe Zhao, Xinyi Ke, Jiayuan Huang, Ziyu Zhang, Yue Wu, Gaoshan Huang, Ji Tan, Xuanyong Liu, Yongfeng Mei*, and Junhao Chu*

Supporting Note 1:

The content of oxide nanomembrane is estimated for a 10 μm thick free-standing nanomembrane-supported MOF film. According to our experiment, the thickness of 200 cycles Al_2O_3 nanomembrane is 18 nm, and therefore the thickness of ZIF-67 film is 9982 nm. According to the literature, the densities of Al_2O_3 and ZIF-67 are 2.8 and 0.94 g cm^{-1} respectively.[8,9] Thus, the fraction of oxide nanomembrane can be calculated by using the equation:

$$Fraction\ of\ Al_2O_3\ (\%) = \frac{18 \times 2.8}{18 \times 2.8 + 9982 \times 0.94}.$$

The fraction of Al_2O_3 nanomembrane is calculated to be 0.53%, which is neglectable in the free-standing nanomembrane-supported MOF film.

Supporting Note 2:

The dynamic simulation of free-standing MOF film was realized by Abaqus, where a two-layer model is adopted and the thickness of each MOF layer is set to be 1 μm. The strain gradient mainly derive from the MOF film/sacrificial layer interface, where tensile strain in lower MOF layer is generated due to the expansion of sacrificial layer. Meanwhile, the opposite side of MOF

film (upper MOF layer) is considered to be completely free. The minimization of the elastic energy after the release of the MOF film leads to re-distribution of the strain in the MOF film and corresponding geometry evolution, i.e., rolling of the film. The simulation result fits the experimental result very well, exhibiting a rolled-up structure with a radius of ~500 μm. Then, the distributions of strain and stress along the thickness of MOF film can also be calculated. The results indicate that the strain increases from 0.35% to 0.08% in MOF film, which is consistent with the strain distribution in rolling model:

$$\varepsilon = c + \frac{y}{R},$$

where ε is strain, y is thickness of MOF film, R is radius of MOF, and c is a constant.

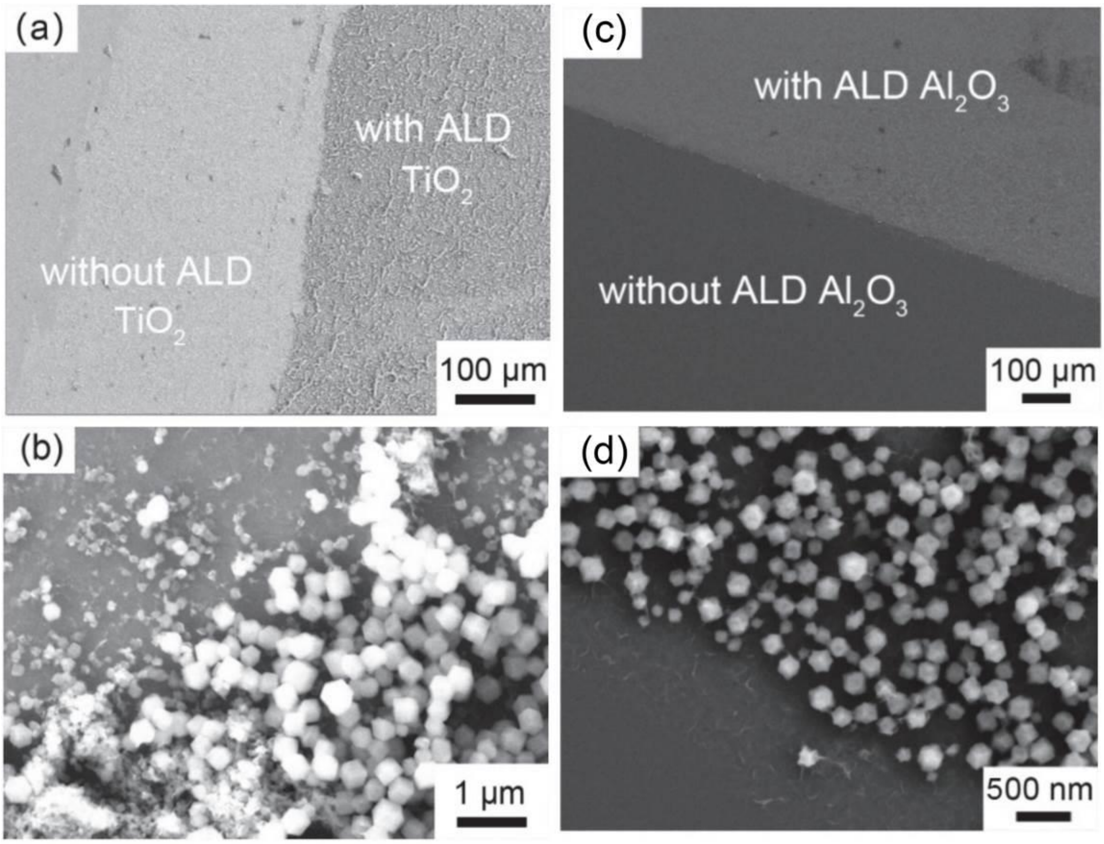

Figure S1. Induction effects of different oxide nanomembranes.

SEM images of MOF film induced by (a) and (b) ALD-TiO_2 nanomembrane and (c) and (d) ALD-Al_2O_3 nanomembrane.

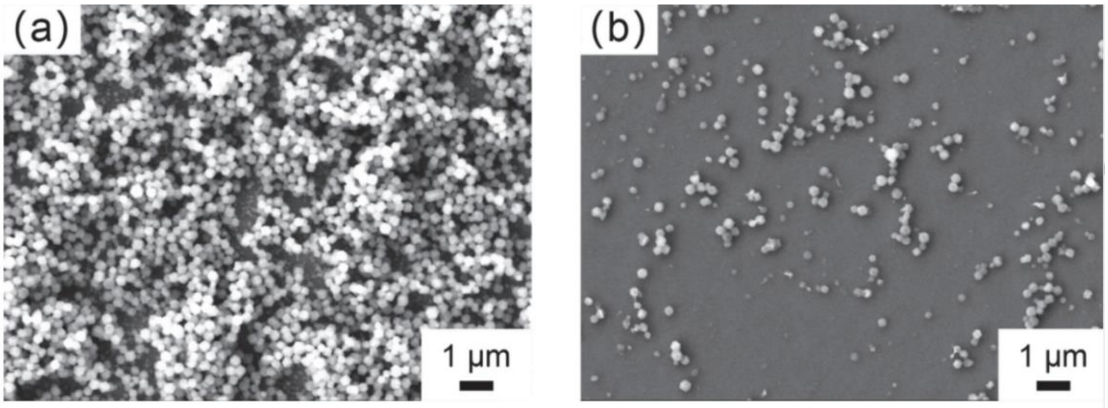

Figure S2. SEM images of MOF film induced by ALD-TiO_2 nanomembrane.

SEM images of MOF film induced by ALD-TiO_2 nanomembrane: (a) before ultrasonication treatment and (b) after ultrasonication treatment.

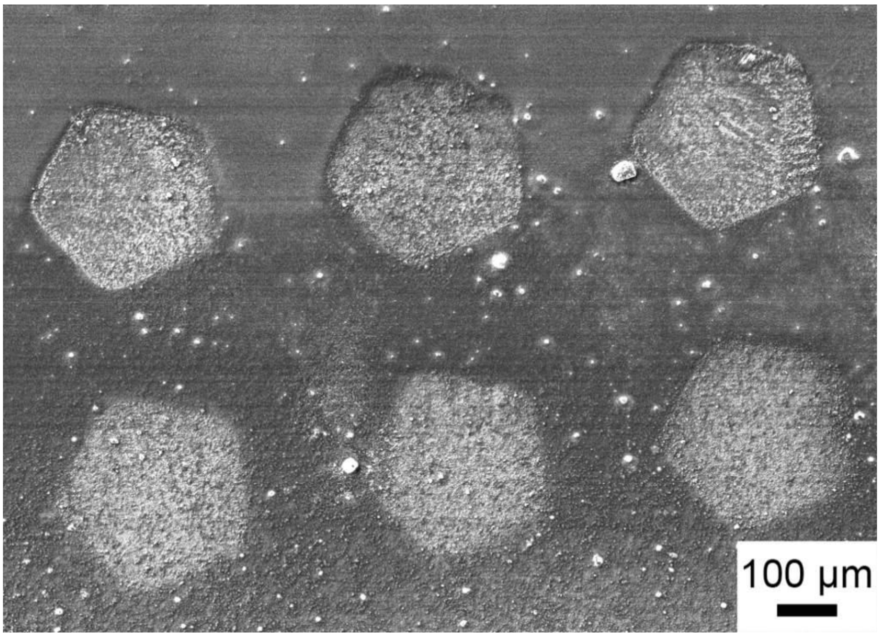

Figure S3. Patterned MOF film.

SEM image of patterned MOF film.

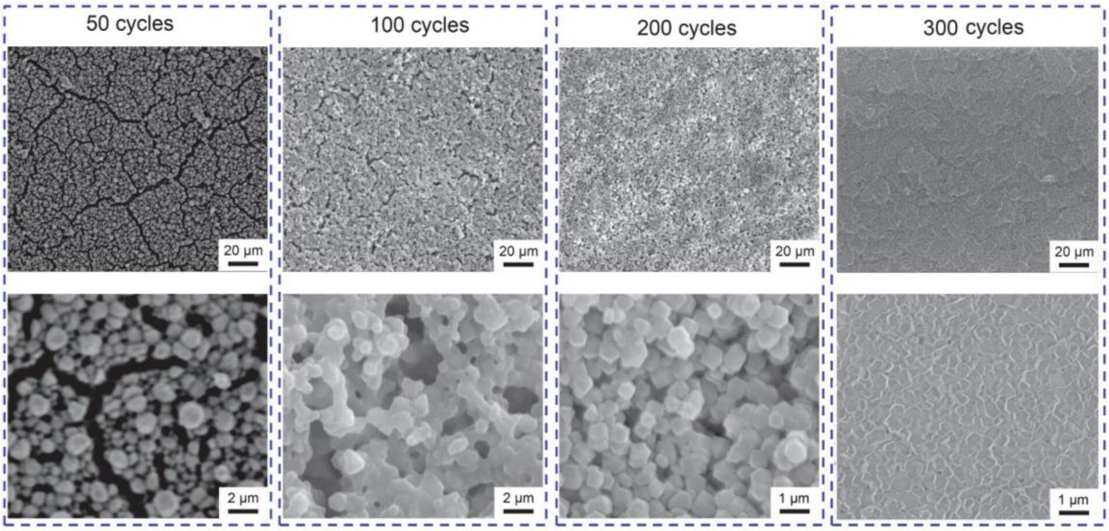

Figure S4. Morphologies of MOF films induced with ZnO nanomembranes with various ALD cycles.

SEM images of MOF films fabricated with the induction of ZnO nanomembranes of different thicknesses.

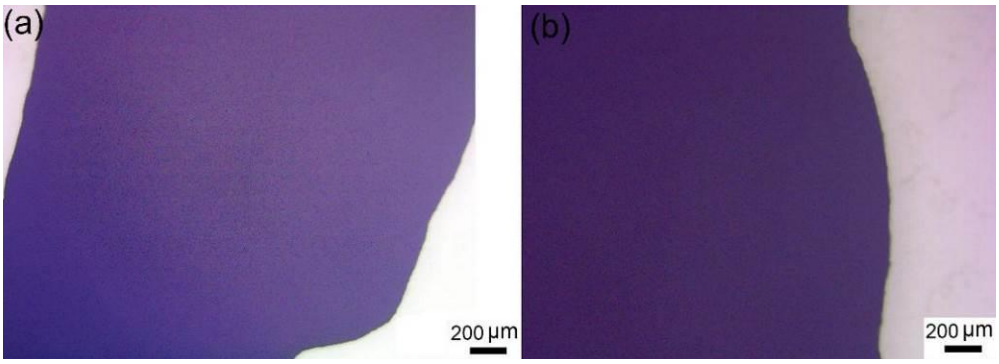

Figure S5. Optical images of the free-standing MOF film.

Optical images of (a) as-prepared free-standing MOF film and (b) free-standing MOF film after being immersed in 0.1 M NaOH for 24 h.

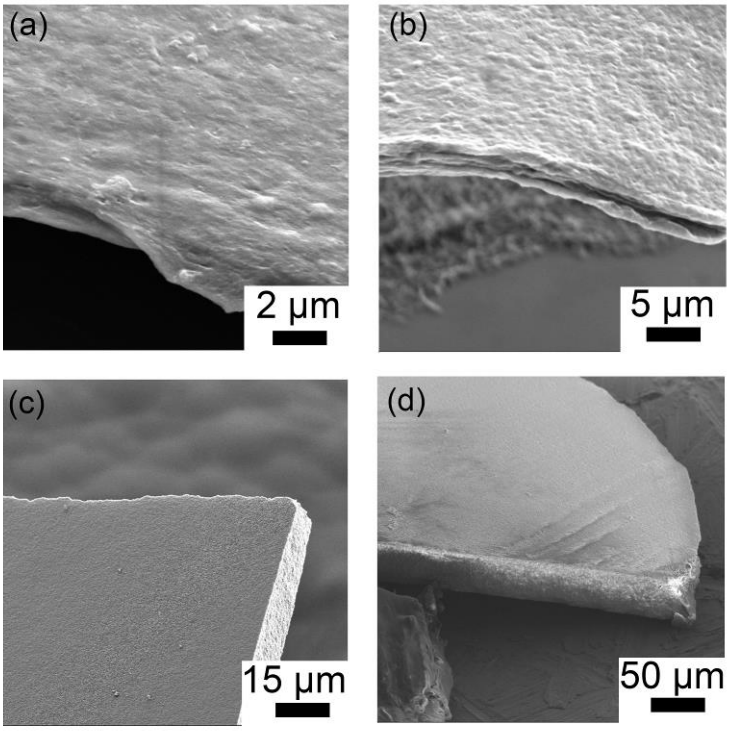

Figure S6. Precise thickness control of MOF films.

SEM images of the free-standing MOF films with various thickness: (a) 2 μm, (b) 5 μm, (c) 15 μm and (d) 50 μm

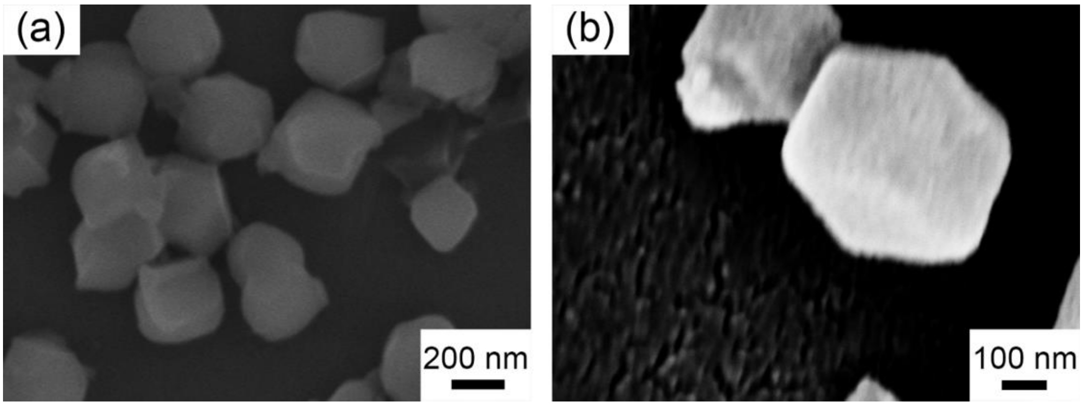

Figure S7. SEM images of MOF particles.

SEM image of ZIF-67 particles conventionally prepared in solution: (a) low-magnification image and (b) high-magnification image.

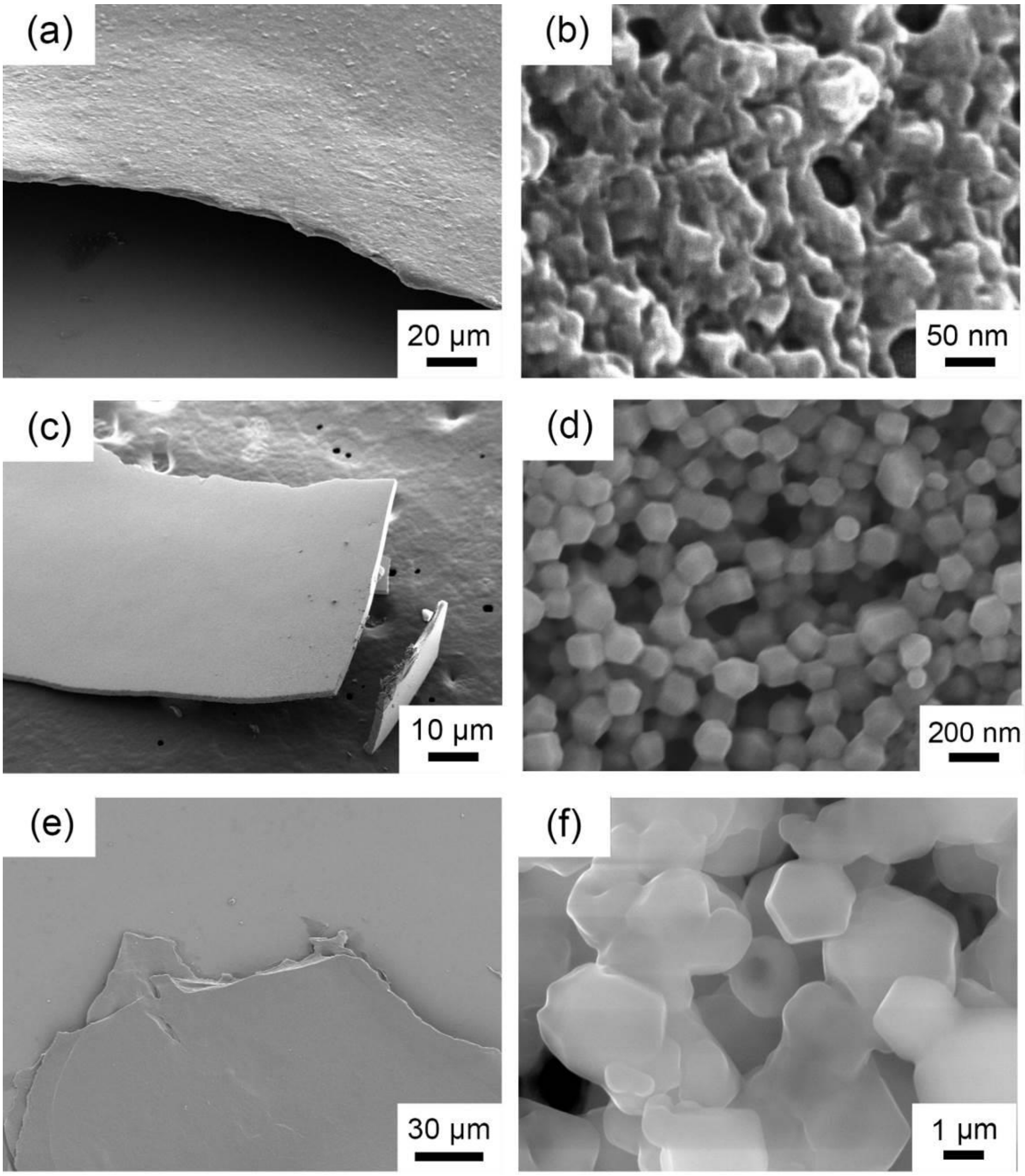

Figure S8. Particle size control in the free-standing MOF films.

SEM image of the free-standing ZIF-67 films consisting of (a) and (b) small particles, (c) and (d) mid particles, and (e) and (f) large particles.

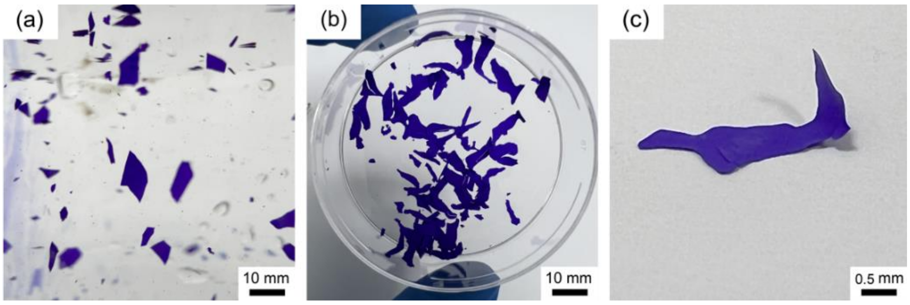

Figure S9. Mass production of free-standing MOF films.

Photographs of the free-standing MOF films. (a) Free-standing films suspended in the water. (b) Large-scale production. (c) Film with self-standing property.

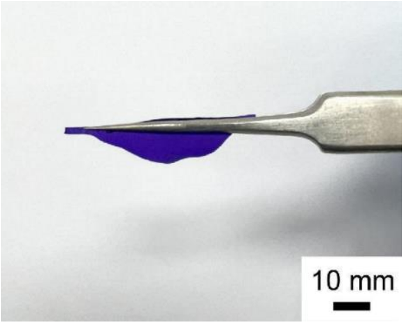

Figure S10. Macro-sized free-standing MOF film.

Optical photograph of a large piece of free-standing MOF film.

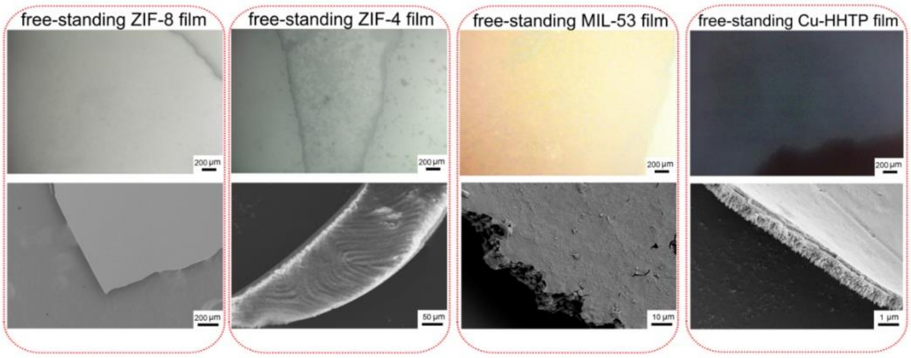

Figure S11. Universality of the fabrication approach.

Photographs (upper row) and SEM images (lower row) of free-standing ZIF-8 film, ZIF-4 film, MIL-53 film, and Cu-HHTP film.

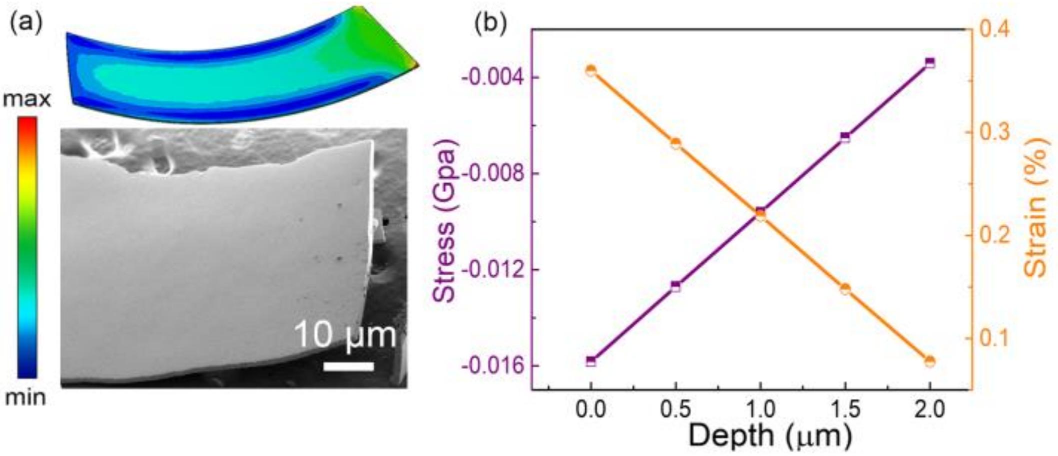

Figure S12. Simulated strain and stress of the free-standing MOF film.

(a) SEM image of the free-standing MOF film as well as the simulated strain distribution.
(b) Simulated strain and stress along the depth of the free-standing MOF film.

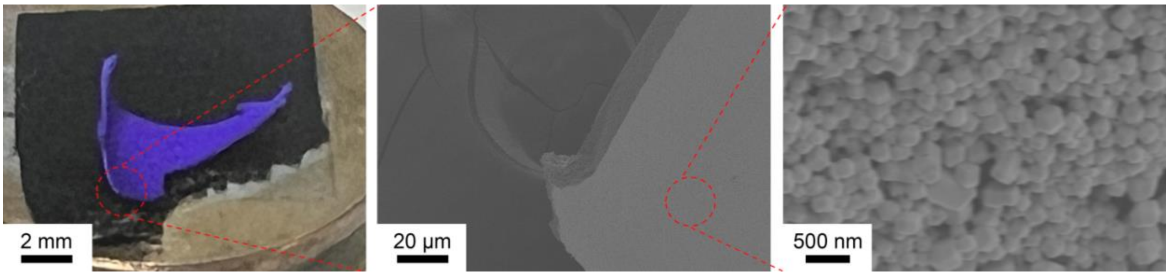

Figure S13. Curved free-standing MOF film.

Optical image and SEM images of curved free-standing MOF film.

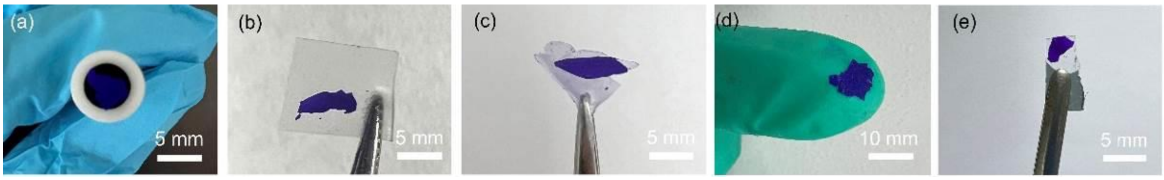

Figure S14. Universality of the transfer process.

Photographs of the transferred free-standing MOFs films on (a) glassy carbon, (b) PDMS film, (c) Eco-flex film, (d) rubber, and (e) indium tin oxide conductive glass.

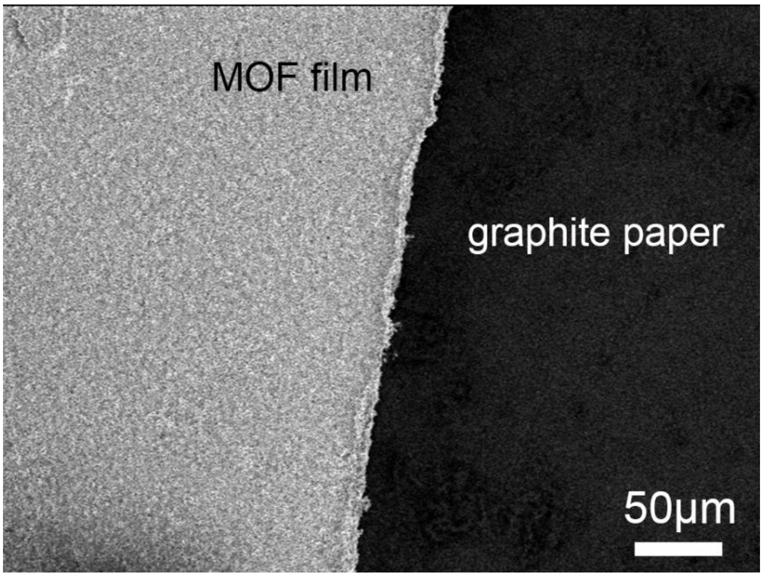

Figure S15. Adhesion of the MOF film on the substrate.

The SEM image of the free-standing MOF film transferred on graphite paper.

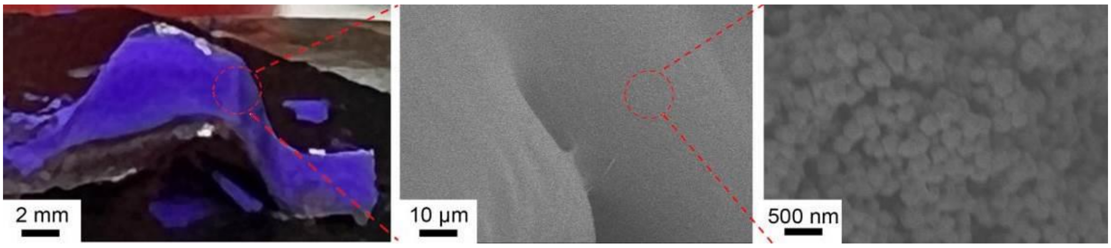

Figure S16. Flexibility of the transferred MOF film.

Optical image and SEM images of transferred MOF film in bending state.

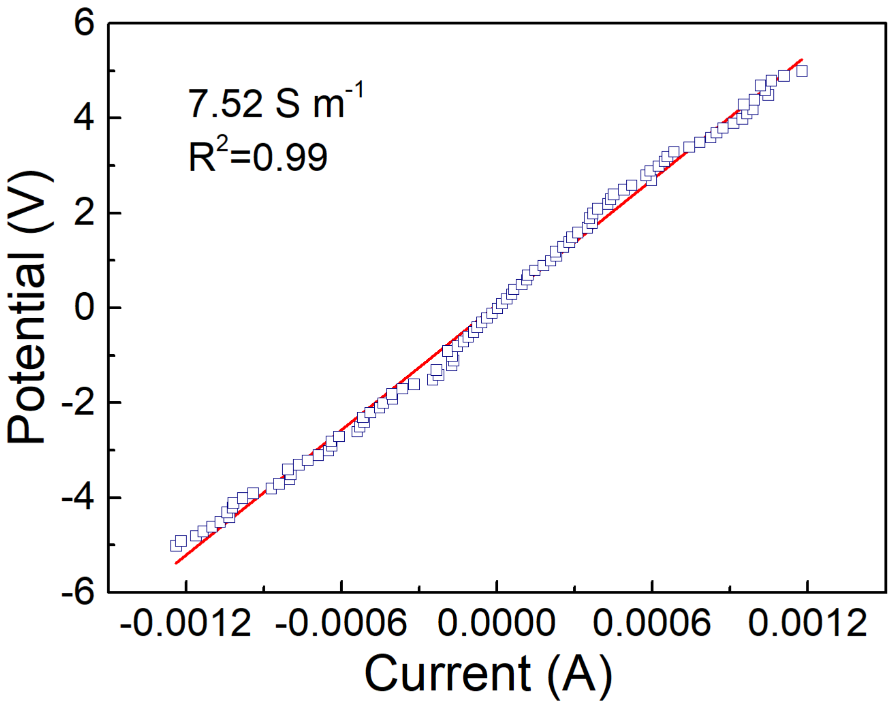

Figure S17. Conductivity evaluation.

I-V curve of the transferred 10 μm MOF film on thin graphite paper (20 μm).

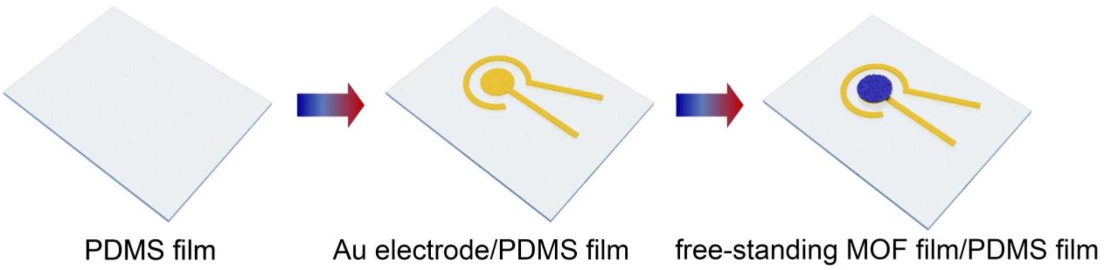

Figure S18. Preparation of the MOF film-based device.

Schematic of the structure of the sensor device.

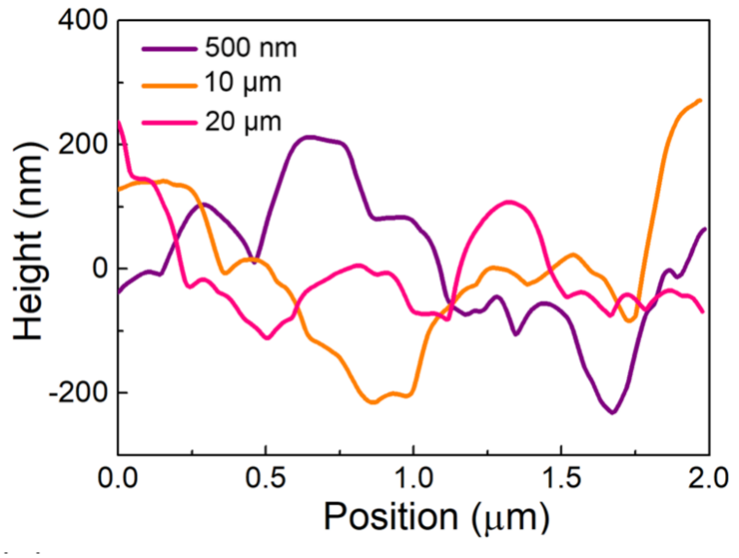

Figure S19. Morphological characterizations of MOF films.

Height-position curves of the free-standing MOF films with various thicknesses.

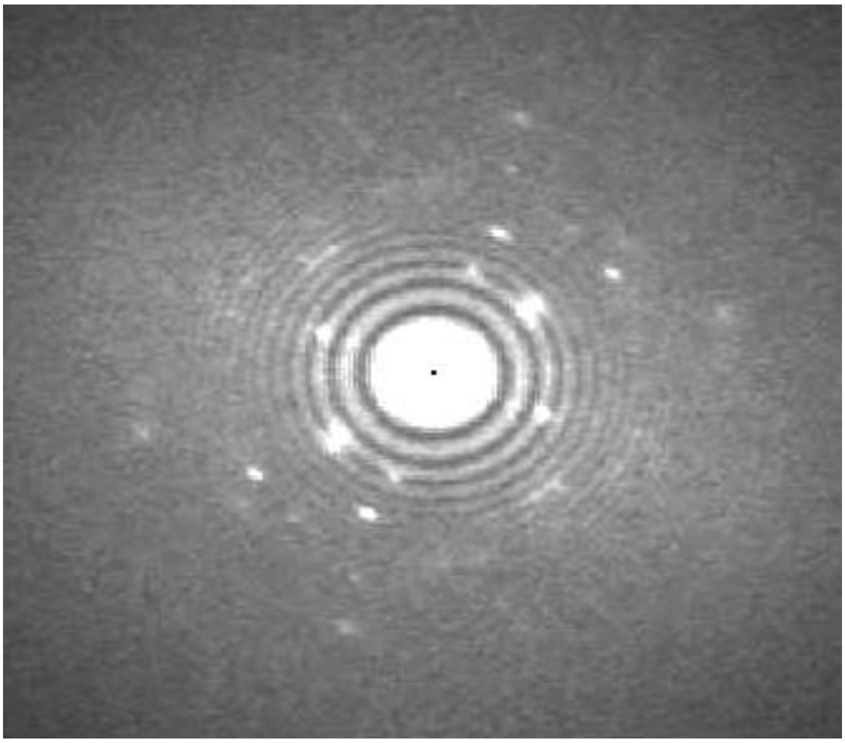

Figure S20. Fast Fourier transform result.

Fast Fourier transform results of TEM image of the free-standing MOF film.

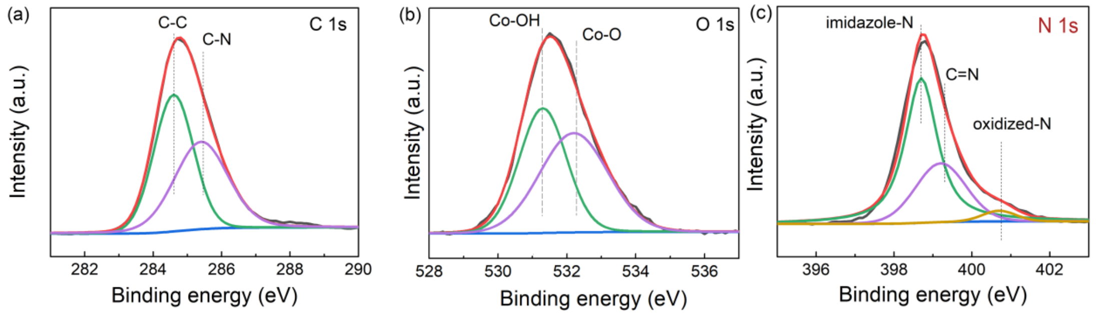

Figure S21. XPS curves of the free-standing MOF film.

High resolution (a) C1s, (b) O1s, and (c) N1s spectra of the free-standing MOF film.

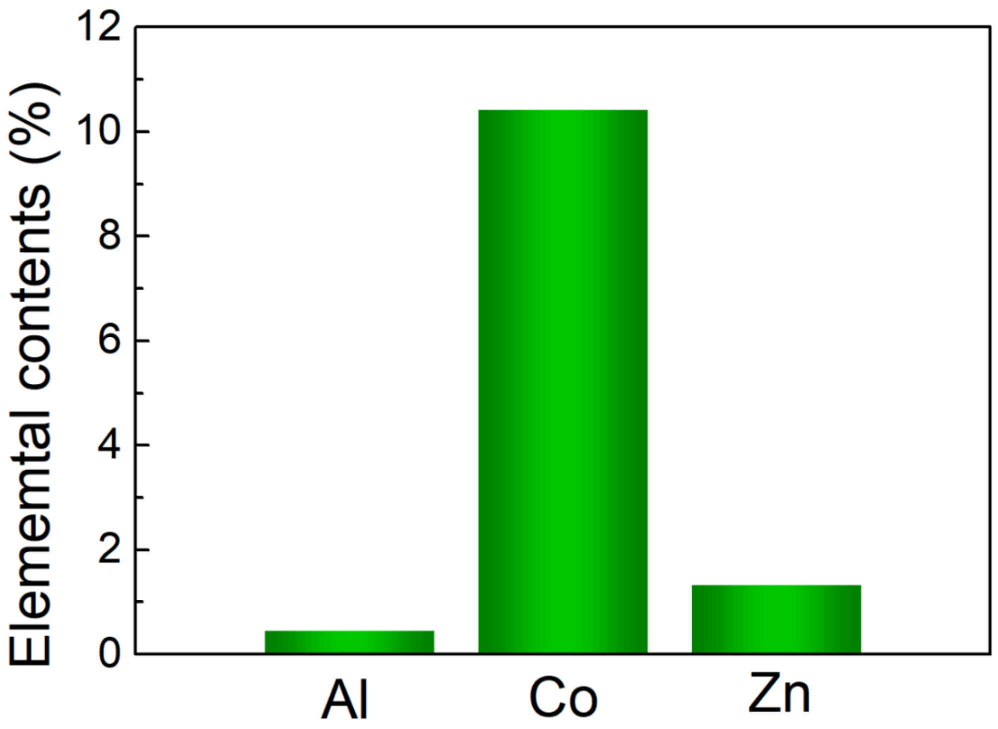

Figure S22. Elemental contents of free-standing MOF film.

Elemental contents of the free-standing MOF film, probed by ICP mass spectrometry.

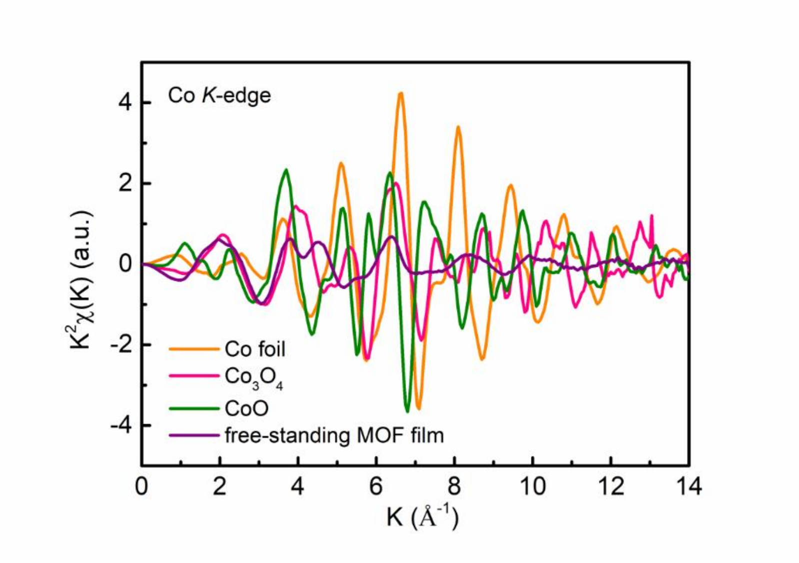

Figure S23. EXAFS results of the samples.

EXAFS of the free-standing MOF film, CoO, Co_2O_3, and Co foil in k space.

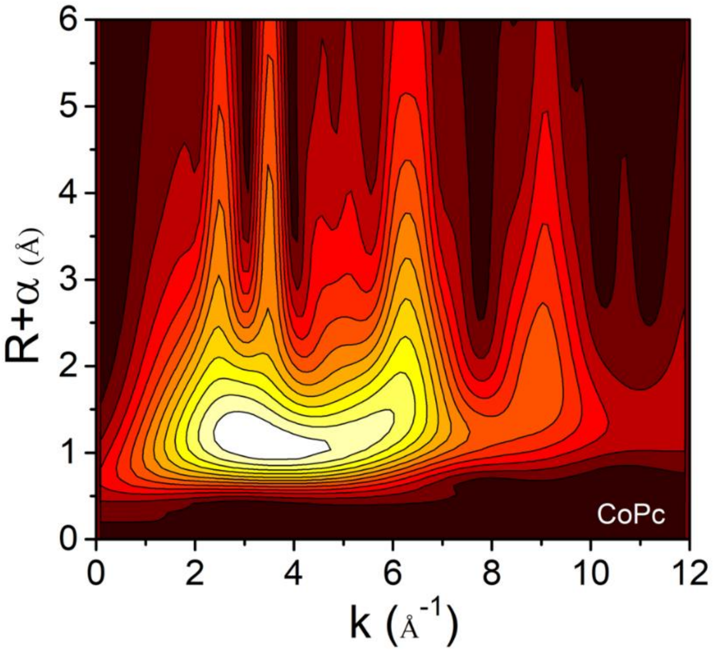

Figure S24. Wavelet transform analysis results.

Wavelet transform analysis of the CoPc.

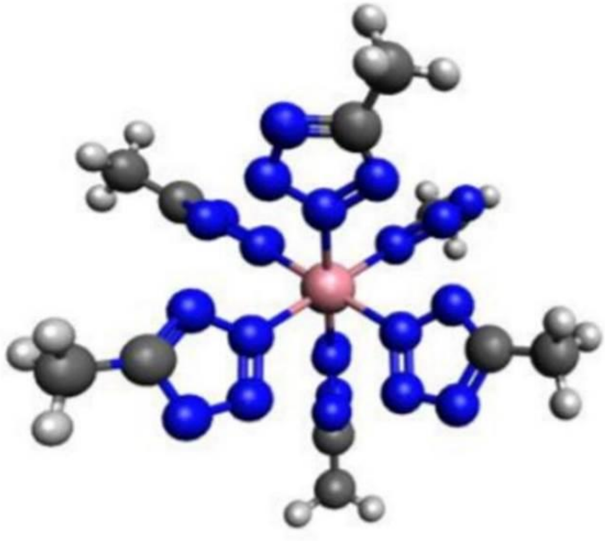

Figure S25. O_h structure model.

O_h structure model of ZIF-67.

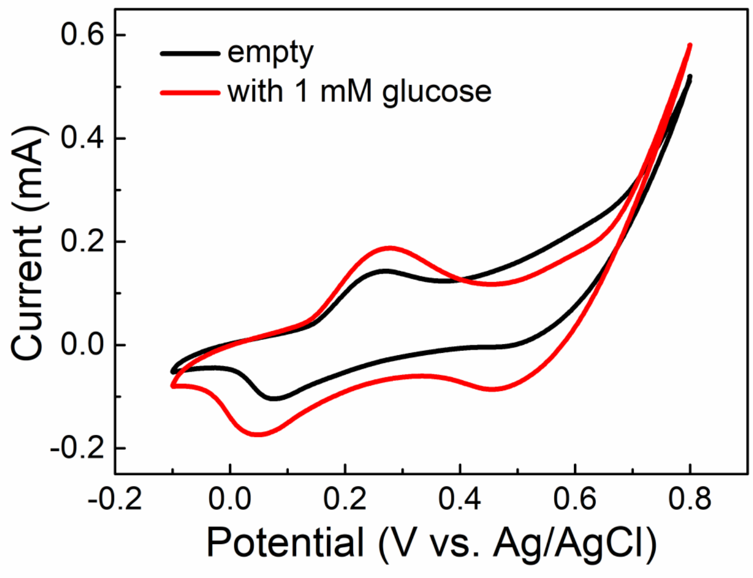

Figure S26. CV curves of the transferred free-standing ZIF-67 film.

CV curves of the transferred free-standing ZIF-67 film with and without 1 mM glucose at a scan rate of 20 mV s^{-1}.

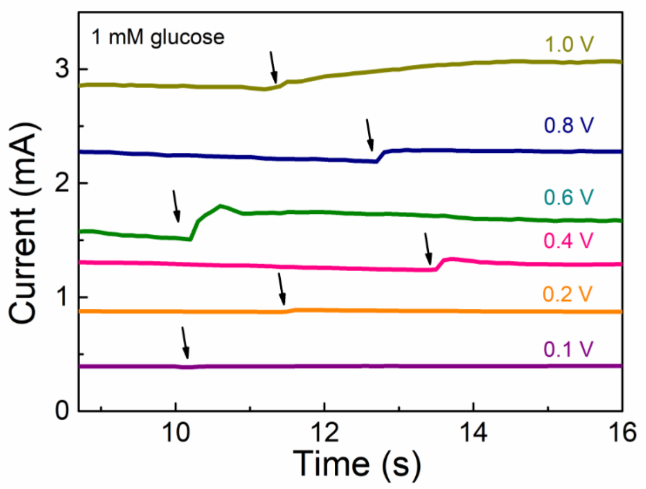

Figure S26. Amperometric response of the transferred free-standing ZIF-67 film.

Amperometric response of the transferred free-standing ZIF-67 film with additions of 1 mM glucose at different potentials.

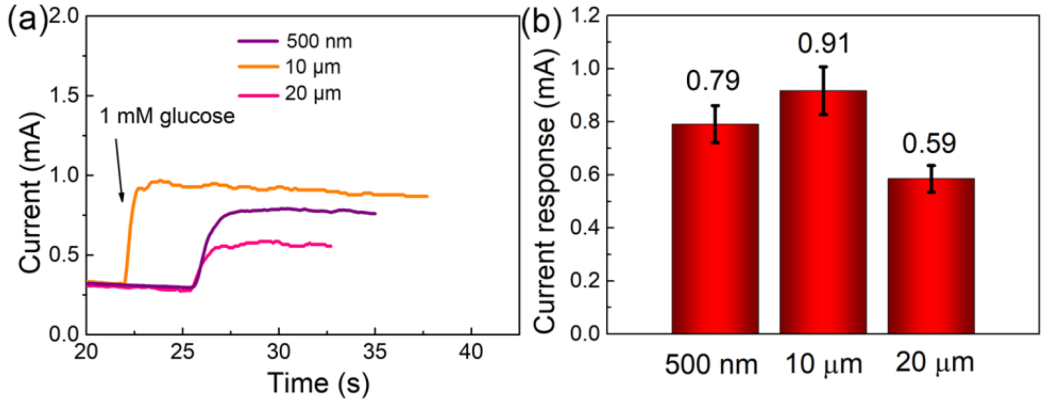

Figure S28. Sensing performance of the transferred ZIF-67 films with various thickness.

(a) Amperometric response of the transferred free-standing ZIF-67 films with various thicknesses when adding 1 mM glucose at 0.6 V. (b) Statistics of the current response of the transferred MOF films towards 1 mM glucose with various thicknesses.

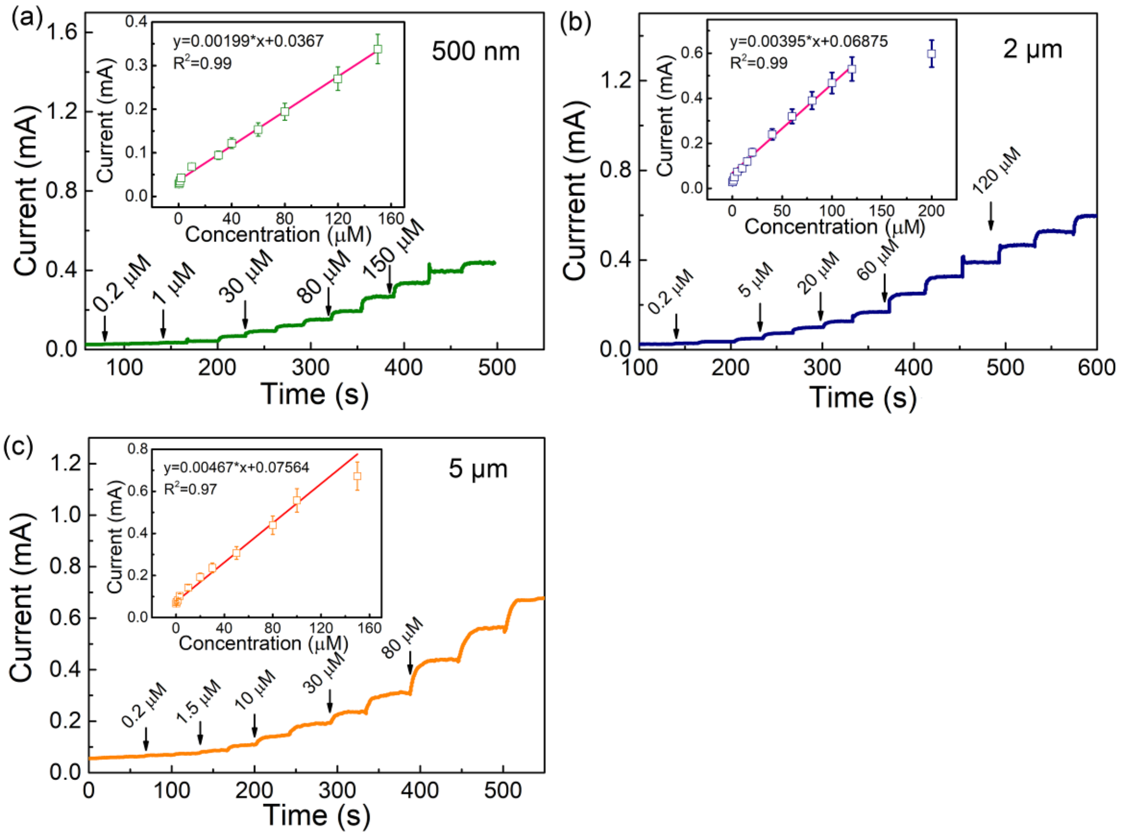

Figure S29. Sensing performance of the thin films.

I-t curves of the transferred free-standing ZIF-67 films with thicknesses of (a) 500 nm, (b) 2 µm, and (c) 5 µm. Glucose solutions with different concentrations is successively added into 0.1 M NaOH electrolyte at a potential of 0.6 V. Insets show the corresponding calibration plots.

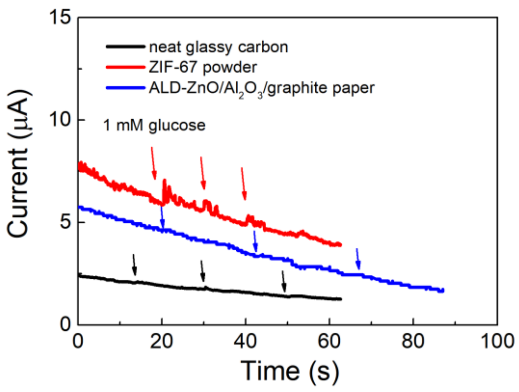

Figure S30. Comparison of the sensing performances.

I-t curves of ZIF-67 particles, ALD prepared ZnO/Al_2O_3 nanomembrane, and neat glassy carbon in 0.1 M NaOH with successive addition of 1 mM glucose.

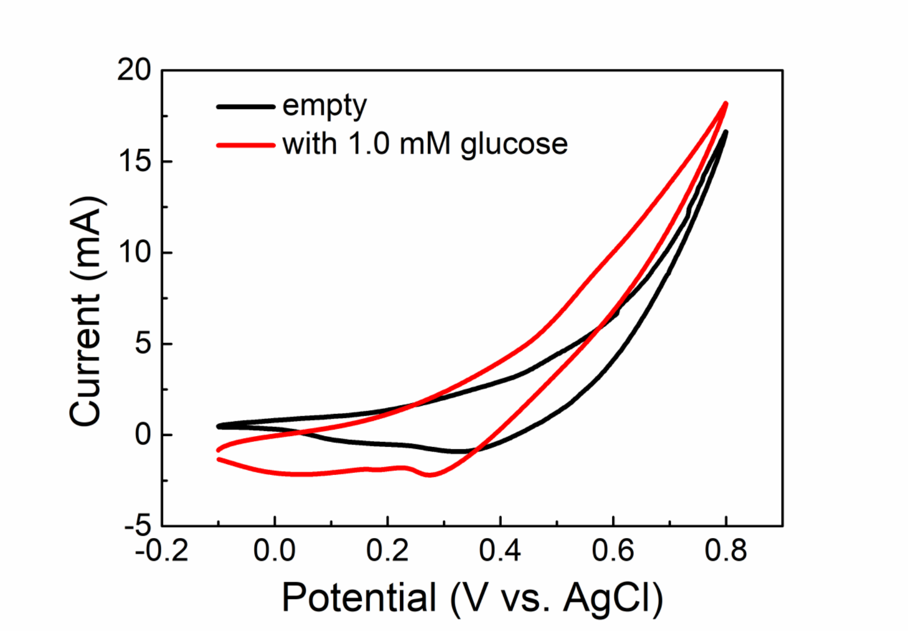

Figure S31. Sensing performance of MOF particles.

CV curves of the ZIF-67 particles electrode with and without 1 mM glucose at a scan rate of 20 mV s^{-1}.

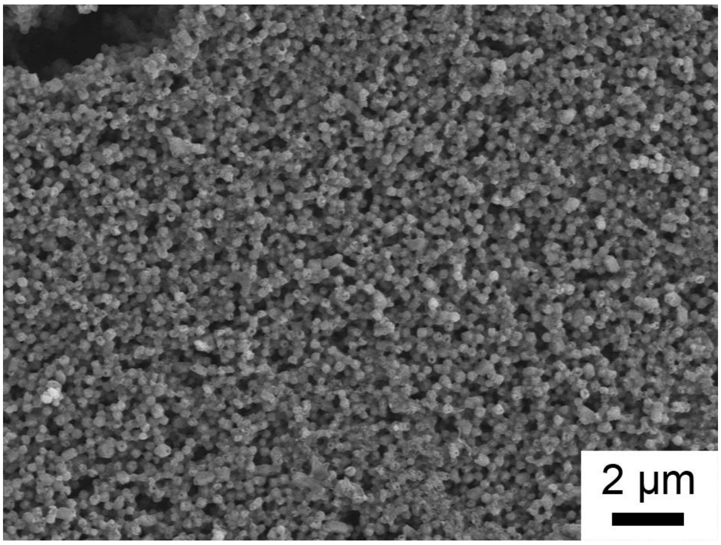

Figure S32. Morphology of the MOF film.

SEM image of the ZIF-67 film directly grown on the surface of thick graphite paper.

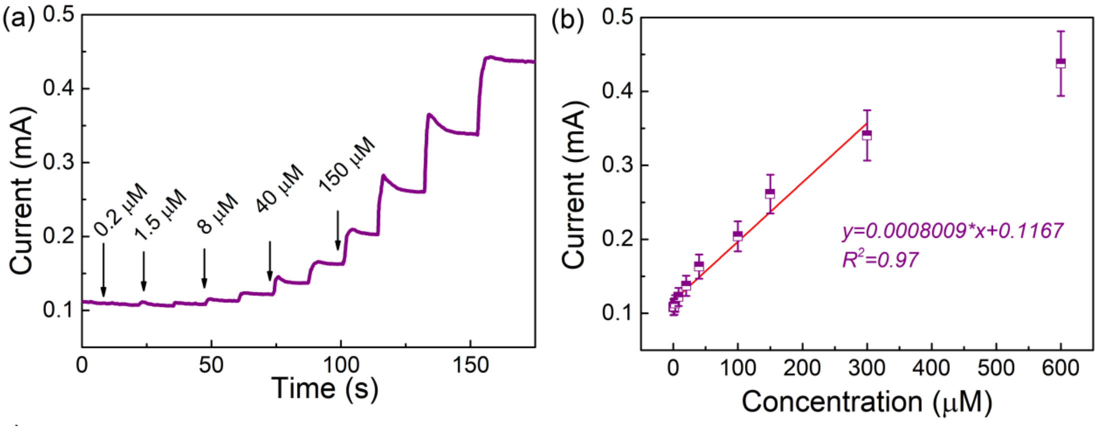

Figure S33. Sensing performance of the MOF film directly grown on thick graphite paper.

(a) I-t curve of the MOF film directly grown on thick graphite paper with successive addition of glucose in 0.1 M NaOH at a potential of 0.6 V. (b) Calibration plot derived from (a).

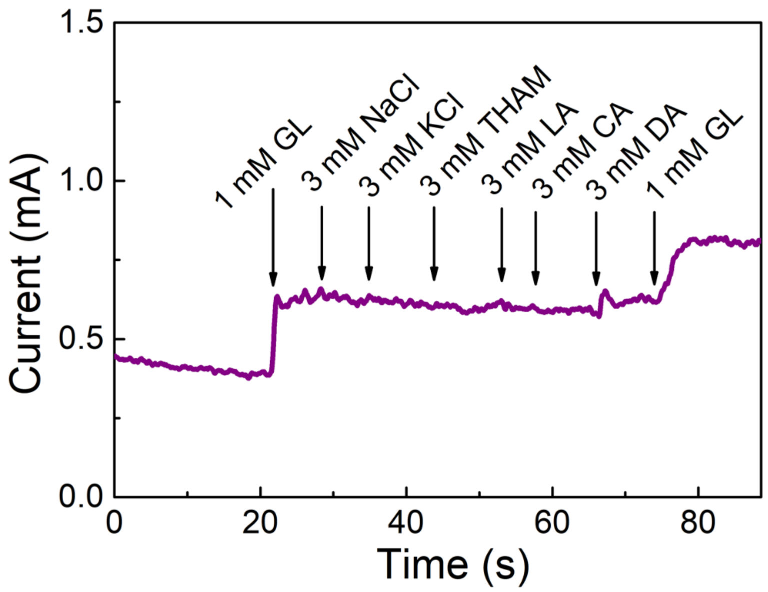

Figure S34. Sensing selectivity of the transferred ZIF-67 film.

I-t curve of the transferred free-standing ZIF-67 film with the successive addition of 1 mM glucose and 3 mM other interferents.

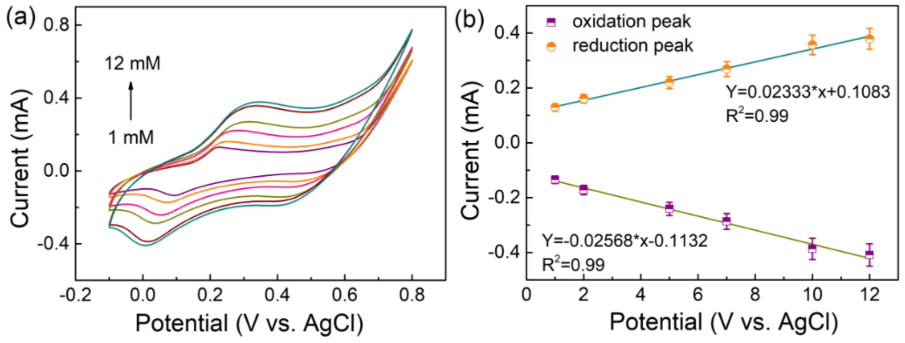

Figure S35. Sensing performance towards glucose with high concentrations.

CV curves of the transferred free-standing ZIF-67 film within the potential range of -0.1 to 0.8 V in 0.1 M NaOH containing glucose with various concentrations at a scan rate of 20 mV s^{-1}. (b) Calibration plots derived from (a).

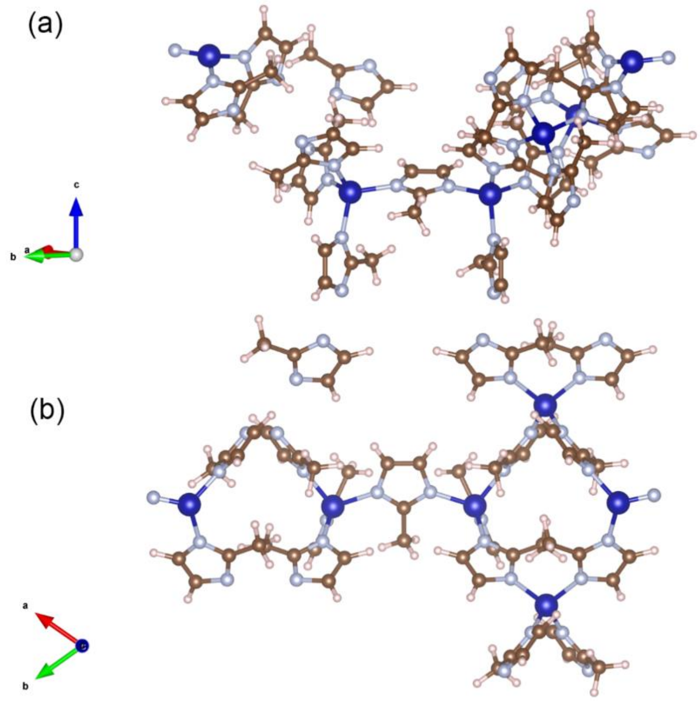

Figure S36. Structure model of the free-standing MOF film.

Structure models of the free-standing ZIF-67 film: (a) side view and (b) top view.

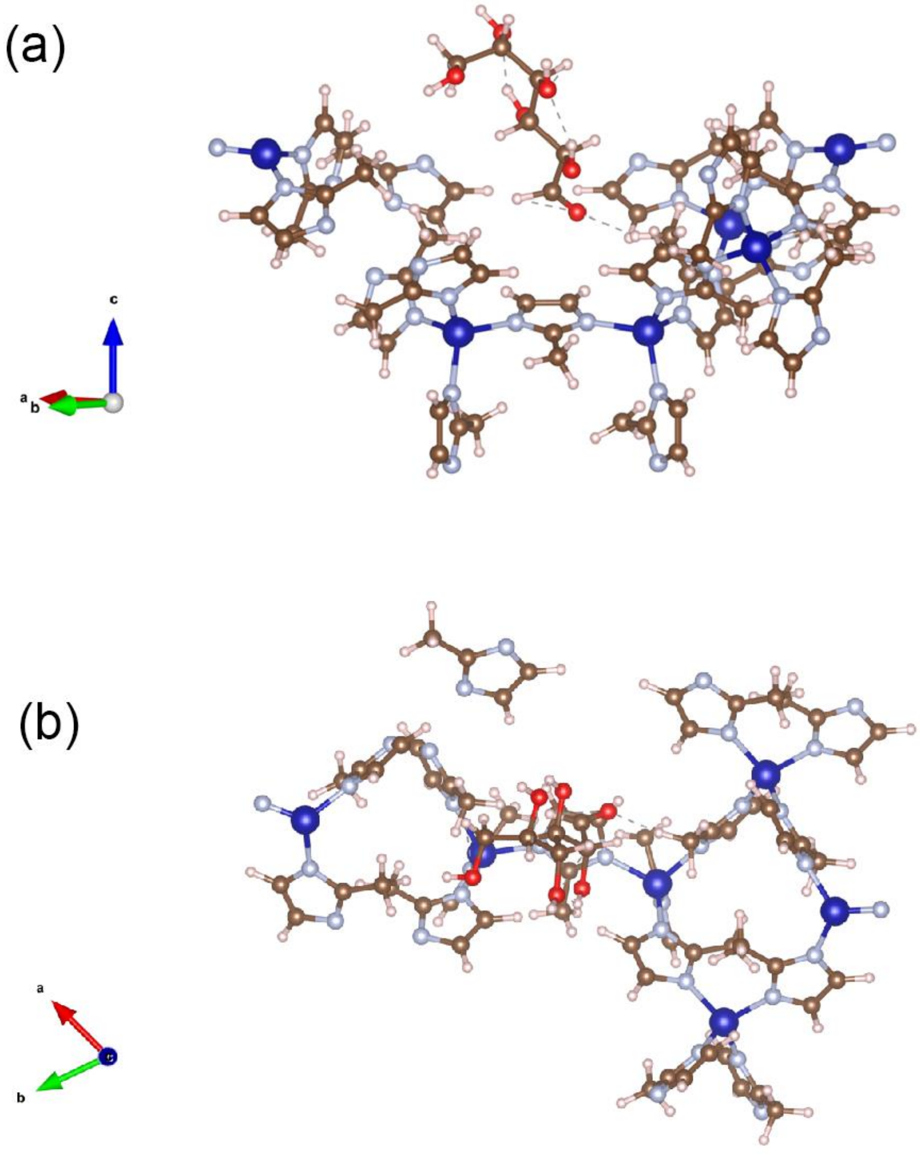

Figure S37. Structure model of the free-standing MOF film after adsorbing glucose.

Optimized structures of glucose adsorbed on free-standing ZIF-67 film: (a) side view and (b) top view.

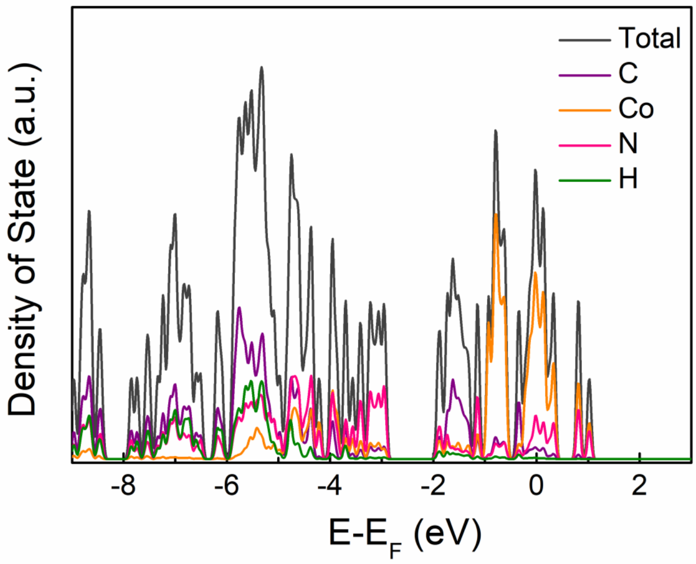

Figure S38. Calculated DOS results.

DOS of free-standing ZIF-67 film.

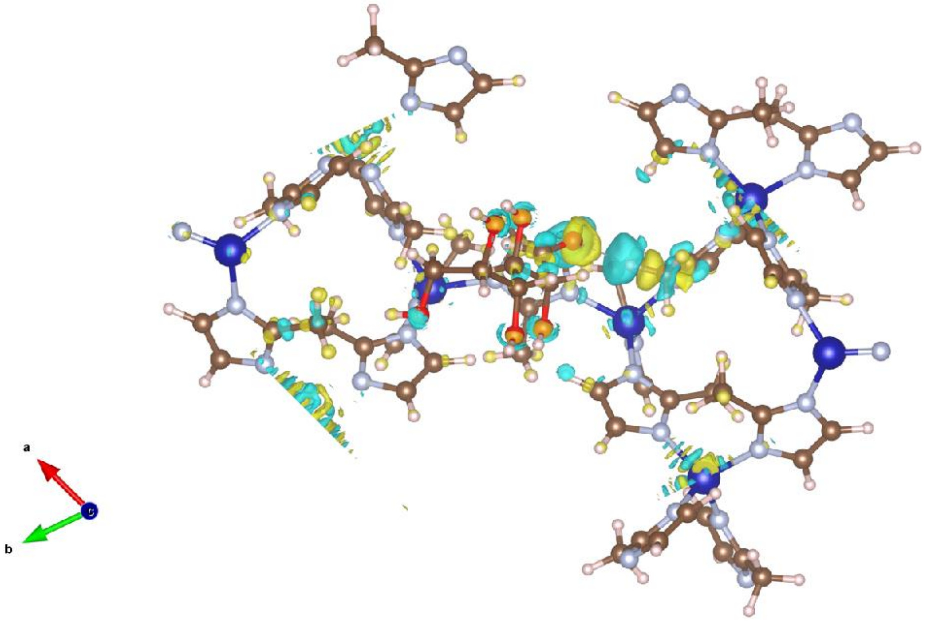

Figure S39. Calculated charge density.

Charge density for single molecule adsorption of glucose (top view).

Table S1 Structural parameters obtained from EXAFS fitting.

Sample	Bond type	N	R (Å)	ΔE_0 (eV)	$\sigma^2 \times 10^3$ (Å^2)
Free-standing MOF film	Co-N	3.9±0.5	2.00±0.01	0.9±1.8	4.4±1.6
	Co-C	7.2±3.8	3.01±0.01	0.1±3.7	15.0±8.8

N, coordination number; R, distance between absorber and backscatter atoms; ΔE_0, inner potential correction to account for the difference in the inner potential between the sample and the reference compound; σ^2, Debye–Waller factor.

Table S2. Comparison of glucose sensing performance of sensors made from transferred ZIF-67 films and other 2D materials published recently.

Sample	Sensitivity (μA mM^{-1} cm^{-2})	LOD (μM)	Linear range	Ref
AuNPs/2D $Ni(OH)_2$	82.71	0.66	0.002-6.0 mM	[1]
2D CuO	2710	0.8	0.001-1.0 mM	[2]
$NiCo_2S_4$@2D-Carbyne	135	34.5	0.02-0.1 mM	[3]
CuO/2D graphene	619.43	0.049	0-3 mM	[4]
2D lamellar Ni-MOF	907.54	0.5	0.5-2665.5 μm	[5]
**Free-standing MOF film on graphite paper**	**4840**	**0.14**	**0.5-300 μM**	**This work**
**Free-standing MOF film on device**	**4035**	**0.18**	**0.5-150 μM**	**This work**

References:

[1] J. Xu, T. Chen, X. Qiao, Q. Sheng, T. Yue, J. Zheng, *Colloids Surf. A* **2019**, 561, 25.

[2] J. C. Bhangoji, C. J. Barile, S. S. Shendage, *ChemistrySelect* **2023**, 8, e202300104.

[3] P. Dhandapani, A. K. S. Petchimuthuraju, S. P. Rajendra, M. S. AlSalhi, S. Angaiah, *ChemPhysChem* **2024**, e202300658.

[4] Q. Li, D. Yang, Y. Gao, Z. Wang, R. Yin, F. Xuan, *Adv. Sensor Res.* **2023**, 2, 2200067.

[5] Q. Zhang, P. Li, J. Wu, Y. Peng, H. Pang, *Adv. Sci.* **2023**, 10, 2304102.
